# Supplementary material for: Is the Parwarish parenting intervention feasible and relevant for young people and parents in diverse settings in India? A mixed methods process evaluation
Source: BMJ Open. 2022 Feb 17;12(2):e054553. doi: 10.1136/bmjopen-2021-054553 (PMC8860057; doi:10.1136/bmjopen-2021-054553)
Supplement: Supplementary data [file bmjopen-2021-054553supp001.pdf]

Supplementary files

Supplementary file 1: Overview of Parwarish curriculum content

Session	Key content
1	Introducing the programme and setting participant goals
2	The value of spending time together to build a positive parent – child relationship
3	Why praise and appreciation build connection – and practicing praise
4	Identifying and talking about emotions in the family
5	Managing anger without hurting others
6	Identifying problems and following steps for problem solving
7/8	The value of saving money and how to make a budget
9	Addressing problems without conflict (2 sessions)
10	Developing family rules and routines to reduce conflict and have fun
11	Ways to save money and make a family savings plan
12	Identifying risks and ways to keep safe in the community
13	How to manage in crisis – safeguards and strategies
14	Getting help and support beyond your household: widening the circle

Supplementary file 2: Qualitative data collection overview

**Participants**	**Data collection**
Facilitators at each site	3 FGD with facilitators (n= 22 people)
Parents and adolescent participants in Parwarish	4 FGD with parents (n = 29) and 4 FGD with adolescents (n= 23)
Online coach session with 3 site coaches and implementation coordinator and researcher	Detailed notes of online session (total 11 pages of typed text)
Facilitators (community-based pairs)	6 individual interviews
Program implementers (Delhi and Agra based)	3 individual interviews
Coaches (located at each of the three field sites)	3 individual interviews
Observations of training, group facilitation in field and coaching sessions	Field notes (total 14 pages of typed text)
Fortnightly logbook documenting highlights and challenges on sessions filled by coaches and shared fortnightly	Fortnightly logbook shared as an email with PN and KM and South African CWB coach
Total number of people contributing to qualitative data	N=86
