# Supplementary material for: Is the Parwarish parenting intervention feasible and relevant for young people and parents in diverse settings in India? A mixed methods process evaluation
Source: BMJ Open. 2022 Feb 17;12(2):e054553. doi: 10.1136/bmjopen-2021-054553 (PMC8860057; doi:10.1136/bmjopen-2021-054553)
Supplement: Author's manuscript [file bmjopen-2021-054553.draft_revisions.pdf]

BMJ Open

BMJ Open is committed to open peer review. As part of this commitment we make the peer review history of every article we publish publicly available.

When an article is published we post the peer reviewers' comments and the authors' responses online. We also post the versions of the paper that were used during peer review. These are the versions that the peer review comments apply to.

The versions of the paper that follow are the versions that were submitted during the peer review process. They are not the versions of record or the final published versions. They should not be cited or distributed as the published version of this manuscript.

BMJ Open is an open access journal and the full, final, typeset and author-corrected version of record of the manuscript is available on our site with no access controls, subscription charges or pay-per-view fees (http://bmjopen.bmj.com).

If you have any questions on BMJ Open's open peer review process please email info.bmjopen@bmj.com

BMJ Open

Is the Parwarish parenting intervention feasible and relevant for young people and parents in diverse settings in India? A mixed methods process evaluation

Journal:	*BMJ Open*
Manuscript ID	bmjopen-2021-054553
Article Type:	Original research
Date Submitted by the Author:	05-Jul-2021
Complete List of Authors:	Mathias, Kaaren; Umea Universitet, Global health and epidemiology; Emmanuel Hospital Association, Community health and development<br>Nayak, Prabhudutt; Emmanuel Hospital Association, Chhatarpur Christian Hospital, Community health and development programme<br>Singh, Pratibha; Emmanuel Hospital Association<br>Pillai, Pooja; Emmanuel Hospital Association, Burans, Herbertpur Christian Hospital<br>Goicolea, I; Umea University, Global health and epidemiology
Keywords:	Public health < INFECTIOUS DISEASES, Non-accidental injury < PAEDIATRICS, Child & adolescent psychiatry < PSYCHIATRY

SCHOLARONE™
Manuscripts

I, the Submitting Author has the right to grant and does grant on behalf of all authors of the Work (as defined in the below author licence), an exclusive licence and/or a non-exclusive licence for contributions from authors who are: i) UK Crown employees; ii) where BMJ has agreed a CC-BY licence shall apply, and/or iii) in accordance with the terms applicable for US Federal Government officers or employees acting as part of their official duties; on a worldwide, perpetual, irrevocable, royalty-free basis to BMJ Publishing Group Ltd ("BMJ") its licensees and where the relevant Journal is co-owned by BMJ to the co-owners of the Journal, to publish the Work in this journal and any other BMJ products and to exploit all rights, as set out in our licence.

The Submitting Author accepts and understands that any supply made under these terms is made by BMJ to the Submitting Author unless you are acting as an employee on behalf of your employer or a postgraduate student of an affiliated institution which is paying any applicable article publishing charge ("APC") for Open Access articles. Where the Submitting Author wishes to make the Work available on an Open Access basis (and intends to pay the relevant APC), the terms of reuse of such Open Access shall be governed by a Creative Commons licence – details of these licences and which Creative Commons licence will apply to this Work are set out in our licence referred to above.

Other than as permitted in any relevant BMJ Author's Self Archiving Policies, I confirm this Work has not been accepted for publication elsewhere, is not being considered for publication elsewhere and does not duplicate material already published. I confirm all authors consent to publication of this Work and authorise the granting of this licence.

For peer review only

Title page

Is the Parwarish parenting intervention feasible and relevant for young people and parents in diverse settings in India? A mixed methods process evaluation

Kaaren Mathias, Prabhudutt Nayak, Pratibha Singh, Pooja Pillai, Isabel Goicolea

Corresponding Author

Kaaren Mathias

Institutional affiliation:

1. Dept of Global health and epidemiology, Universitet Umea, Sweden
2. Emmanuel Hospital Association, New Delhi, India

169 Southampton St, Sydenham, Christchurch, New Zealand 8023

Email kaaren.mathias@umu.se

Ph +64 20 41497530

Co-authors

Prabhudutt Nayak

Emmanuel Hospital Association, Chhatarpur, India

Pratibha Singh

Emmanuel Hospital Association, New Delhi, India

Pooja Pillai

Emmanuel Hospital Association, Herbertpur, India

Isabel Goicolea

Dept of Global health and epidemiology, Universitet Umea, Sweden

Keywords

For peer review only

India, parenting, family violence, process assessment, community

Word Count = 4459

For peer review only

For peer review only - http://bmjopen.bmj.com/site/about/guidelines.xhtml

Is the Parwarish parenting intervention feasible and relevant for young people and parents in diverse settings in India? A mixed methods process evaluation.

Kaaren Mathias, Prabhudutt Nayak, Pratibha Singh, Pooja Pillai, Isabel Goicolea

Abstract n=296 words

Objective

To assess the feasibility, acceptability and relevance of the Parwarish, a positive parenting intervention (adapted from PLH-Teens) in three diverse settings in India.

Design

This mixed methods study used the Medical Research Council framework for process evaluations of complex interventions.

Setting

This study was set in disadvantaged communities in urban Agra, rural Uttar Pradesh and tribal Jharkhand in India.

Participants

Data was collected from 86 facilitators, implementers, parents and teens who participated in the Parwarish intervention among 239 families.

Intervention

Couples from target communities facilitated groups of parents and teens over the 14 -module structured, interactive Parwarish intervention which focused on building communication, reducing harsh parenting and building family budgetting skills.

Outcome measures

We assessed relevance, acceptability and feasibility of the intervention using mixed methods. Qualitative data collected included semi-structured interviews and focus group discussions with implementers, facilitators, parents and young people that were transcribed, translated and thematically analysed to develop themes inherent in the data. Quantitative data which assessed attendance, fidelity to the intervention and facilitator training and coaching were analysed descriptively.

Results

Findings were grouped under the three domains of facilitation, community engagement and program support with the following seven themes: 1)community-based facilitators increased contextual validity of the intervention; 2) gender relations influenced Parwarish implementation, but were also influenced and transformed by Parwarish; 3) facilitator responsiveness to group concerns increased participation; 4) participation gathered momentum; 5) Parwarish's strong core and porous periphery allowed adaptations to local contexts; 6) Technology that included Skype and Whats app enhanced implementation and 7) critical reflection with community trained coaches strengthened facilitation quality and program fidelity.

For peer review only - http://bmjopen.bmj.com/site/about/guidelines.xhtml

Conclusion

This study found Parwarish engaging, feasible and acceptable in three diverse, low-income communities, although constrained by patriarchal gender relations. It paves the way for larger-scale implementation in other South Asia settings.

Strengths and limitations of this study

- This study is set in three diverse settings
- Research conducted by programme implementers allows close-up understanding of the context
- There is a risk of social desirability bias in responses from participants due to research being conducted by some programme implementers.
- The 'real world' study design increases generalisability of the findings
- The 'real world' study design meant the intervention was adapted to different contexts, reducing fidelity to the programme.

Protocol

This study has no study protocol.

Acknowledgements

We thank the Jacob Gwal and Englina Ela Rani Tirkey (coach) with the Khunti team, Somesh Singh and Deepika Tikvah (coach) with the Agra team and Jyotsana Saha (coach) with the Robertsganj team for all their enthusiasm and diligent work implementing Parwarish and supporting with data collection for this study. We are also thankful for the support in adaptation of Parwarish to Jeet Bahadur, Prerana Singh, Amanda Raymond and the Experts by Experience group of mothers and others in the Community Health and Development programme of the Emmanuel Hospital Association.

For peer review only

For peer review only - http://bmjopen.bmj.com/site/about/guidelines.xhtml

Introduction

Young people in India experience high rates of physical, sexual and emotional abuse as well as neglect (1, 2) with a recent study estimating that 70% lifetime prevalence of maltreatment among adolescent girls in Delhi (3). India has more adolescents than any other country, and the millions affected by maltreatment risk current and life-long problems with physical and mental health, well-being, education and employment (1, 4, 5).

There is growing evidence that interventions focusing on positive parenting can reduce abuse of young people and children (6-8). Further, the most effective programmes are participatory, address underlying risk factors like gender relations and support reduced non-violent behaviours, greater communication and shared decision making among family members, yet globally, 10% or fewer of adolescents can access violence prevention programs.(4, 9).

Parenting for Lifelong Health is a positive parenting group intervention for parents and adolescents, that builds on social learning and parent management training principles seeking reduce all types of violence in families (10). Other publications detail PLH-Teens' development, theory, and effectiveness in South Africa (7-11). PLH-Teens was adapted for the Indian setting by Emmanuel Hospital Association and named Parwarish (meaning 'nurturing' in Hindi). This paper reports a process evaluation of Parwarish in three diverse communities in Central and North India.

Process evaluations are valuable to assess the fidelity and quality of implementation, to clarify causal mechanisms and identify contextual factors associated with variation in outcomes and to build interventions that are more generalisable, relevant, adaptable and ultimately scalable (12-14). Examining feasibility assesses whether an intervention is relevant, acceptable and how it can be adapted contextually (15). There is very limited research documenting implementation of family violence prevention programmes, particularly in community and low- and middle-income settings (4, 5, 16). Many describe the urgent need for studies that examine context and implementation processes in order to better develop interventions that reduce violence in communities, particularly in low- and middle-income settings (4-6, 14, 16, 17).

We aimed to address two questions as we implemented Parwarish in three diverse setting in North India: firstly, what was the feasibility of implementing the Parwarish intervention?; secondly, how relevant and acceptable was Parwarish for community facilitators, parents and adolescents?

Methods

Conceptual framework

This mixed methods study merged two evaluation frameworks, namely process and feasibility evaluation, as we sought to understand how the intervention was delivered, and also whether it was feasible and acceptable. We followed the Medical Research Council framework for process evaluation of complex interventions examining how the intervention was delivered, and the emerging adaptations of the intervention related to context and actors (12). Additionally, we assessed feasibility (practicality, implementation, adaptations, acceptability and demand) (15).

Setting of the intervention

This study examined Parwarish implementation in three diverse settings: In Agra, a large city in Uttar Pradesh, in Robertsganj, a small town in Sonbhadra district, Uttar Pradesh and tribal Khunti, Jharkhand.

For peer review only - http://bmjopen.bmj.com/site/about/guidelines.xhtml

Parwarish was implemented by teams from the Community development and Health programme (CHDP) of the Emmanuel Hospital Association in North India. India ranked 140th globally in the latest Global Gender gap report (18) and this patriarchy is evidenced by the lower literacy of women and sex ratios at birth. An overview of the demographic profile of the study areas compared with national Indian data is provided in Table 1.

Table 1. Socio-demographic profile of the three districts where Parwarish was implemented, with reference data for India nationally (19)

Indicator	National - India	Agra City - UP	Sonbhadra district UP	Khunti district, Jharkhand
Total population (million people)	1,200	4.41	1.86	0.53
% population rural	72.2	54.2	83.1	91.5
% population under 18 years (State)	34.9	33.7	33.7	33.5
% population Scheduled castes/Scheduled Tribes	25.2	22.6	14.9	77
Sex ratio (female to 1000 males)	940	868	918	997
Literacy (% literate female)	65.5	61.2	52.1	53.7
Literacy (% literate male)	82.1	80.6	74.9	74.1
Prevalence of gender-based violence (% at State level)	40	47	47	30 – 50

For peer review only

For peer review only - http://bmjopen.bmj.com/site/about/guidelines.xhtml

Intervention

Intervention adaptation

Parwarish was adapted for India from PLH-Teens(10), with a parenting expert group from the Emmanuel Hospital Association[1] (EHA) working in consultation with a group of mothers of adolescents (experts by experience) from disadvantaged communities in Dehradun, Uttarakhand. Together they reviewed both the superficial structures (language, role plays) as well as cultural content. with two key outputs being the Parwarish facilitator manual and Parwarish family workbook, both produced in plain-language English and Hindi.

Intervention content

Parwarish seeks to reduce harsh parenting and all violence within families through new attitudes and skill building between parents and adolescents. Content includes spending quality time together, communicating positively, managing strong emotions like anger and finding safe support when needed as well as problem solving, conflict resolution and finance skills, including how to manage a household budget and uses role-plays, activity-based learning and home rehearsal and activities building on social learning (10, 11, 20, 21).

Facilitator selection and training process

Parwarish was implemented by pairs of community facilitators with the following criterion for facilitation selection:

- parents of adolescents who were resident in the target community.
- represent an equal mix of genders willing to work as a pair in facilitation (over half of facilitators worked as a married couple).
- trusted and accepted as a leader by the community.
- effective communicators.
- had obtained at least 10th class pass and were fluent in the local dialect or language.

Parwarish facilitation

Trainers from PLH-Teens South Africa facilitated a 10-day course for Parwarish facilitators, and a 3-day training for coaches (total n=25 participants). The facilitators then led the 14 Parwarish modules with groups of parents and teens with meetings of 1.5 to two hours and encouraged participants to complete the weekly activity to try at home E.g., family eats dinner together. Home visits with those who missed a session review the topic for the week.

In each site, a local 'coach' supported facilitators with weekly meetings reflecting on their facilitation while a fortnightly coach-the-coaches meeting was led online with someone from PLH-Teens South Africa.

Recruitment of target communities and participants

Community health and development team members from EHA recruited participants by informing them about Parwarish and inviting participation. Inclusion criteria were:

---

[1] EHA is a non-profit organisation with over 2000 staff, working across North and North-East India with charitable hospitals, community health and development programmes.

For peer review only - http://bmjopen.bmj.com/site/about/guidelines.xhtml

- at least one adolescent (aged 12 – 18 years) in the household
- resident in the target community
- at least one parent/ carer and one adolescent available for weekly sessions.

A total of 239 of 245 families invited agreed to join the programme (97.6%). The socio-demographic profile of community participants profiled in Table 2 demonstrates participants were highly disadvantaged, where over 75% of mothers hadn't completed primary schooling.

Table 2. Socio-demographic profile of Parwarish participants

(N=239 families)	Agra (%)	Robertsganj (%)	Khunti (%)	Parent	
	Adolescents/household			Mother	Father
**Female**	68 (52.7)	36 (60.0)	32 (64.0)	-	
**Age in years (Mean)**	13.3	13.3	14.0	36.8	40.2
**Caste (Indian census classification) :**					
Scheduled caste or tribes	110 (85.3)	51 (85)	50 (100)	199(83%)	
Other backward castes	19 (14.7)	9 (15)	-	37 (15%)	
General	-	-	-	3 (1.3%)	
**Religion:**					
Muslim	11 (8.5)	3 (5)	-	14 (6%)	
Hindu	117 (90.7)	57 (95)	3 (6)	167 (70%)	
Christian	-	-	21 (42)	29 (12%)	
Other religion	-	-	26 (52)	29 (12%)	
**Education:**					
Never been to school	31 (24.0)	-	-	135 (56%)	83 (35%)
Class 5 completed	46 (35.7)	32 (53.3)	23 (46)	48 (20%)	48 (20%)
Class 10 completed	44 (34.2)	48 (46.6)	20 (40)	43 (18%)	83 (35%)
Class 12 completed	8 (6.2)	-	3 (6)	2 (1%)	7 (3%)

Data collection

In-depth interviews and focus group discussions (FGDs) were collected face-to-face in the community by KM, PS and PN in Hindi with 86 purposively sampled facilitators, parents and young people and continued until data saturation was reached. They lasted between 30 and 70 minutes and were recorded, translated and transcribed into English. Interview guides were developed by authors and piloted in the community. They asked about implementation processes, acceptability and feasibility as well as focus group discussions (FGD) with parents, adolescents and facilitators. Additional field notes and free text was recorded in coach and facilitator documents. Supplementary Table 1 provides an overview of the qualitative data collection.

Coaches also filled a paper register at each site on the following measures:

- Facilitation and fidelity: 14 of 14 sessions self-reported by facilitators using a 5-point checklist on: physical exercise, emotional check in, main teaching topic, role play and completion of home activity.

For peer review only

For peer review only - http://bmjopen.bmj.com/site/about/guidelines.xhtml

- Facilitation and fidelity: 4 of 14 sessions for each Parwarish group, filled by the coach or a researcher assessing the five aspects above and two additional aspects, namely: facilitator engagement, and completion of registers.
- Attendance of facilitators and coaches at trainings and coaching: attendance register.
- Attendance of parents and adolescents: attendance register
- Completion of assigned home activities: register.
- Home visits completed by facilitators: register.

Analysis

Qualitative interviews and written documents were read and re-read and analysed with thematic analysis developed by Braun and Clarke (22). Four transcripts were inductively coded by PN and KM and these codes were reviewed to develop a coding framework, paying attention to implementation processes, feasibility and barriers and enablers of the intervention. Remaining transcripts were then coded with this framework by KM and PN, who collaboratively developed categories and then themes, looking for patterns inherent in the data. Quantitative data was analysed descriptively and tabulated however given geographically dispersed field sites data was not member checked with parents and children in communities.

Stance and ethics

This study was conducted by participant researchers who had all lived in India for at least 24 years and spoke fluent Hindi. KM, PS, PP (women) and PN (man) were employed by the implementers Emmanuel Hospital Association. KM (PhD) facilitated adaptation of PLH-Teens to Parwarish and led meetings with experts by experience. PS and PN (MPH and MSW) supported Parwarish implementation at a high level. PP (B Med Sci) and IG PhD) are women who work in India and Sweden respectively.

Patient and public involvement statement

Parents were involved in this study in three key ways. Firstly, parents were the impetus for this Parwarish intervention, having requested EHA team members in Dehradun, Agra and Khunti to support them to build their skills in raising young people. Secondly, parents were part of the process of adaptation of the intervention. A group of parents who resided in informal communities in Dehradun were consulted as “Experts by experience” in reviewing and suggesting contextually relevant adaptations of Parwarish. Thirdly, parents from intervention target communities were identified and trained as facilitators of this intervention and thus were also involved in recruiting participants, and collecting data linked to implementation required by facilitators such as the highlights and lowlights register. Parents in communities will also be involved in sharing findings at dissemination meetings planned after COVID-19 restrictions are lifted later in 2021 using community meetings and reviewing a plain-language Hindi summary of study findings.

For peer review only - http://bmjopen.bmj.com/site/about/guidelines.xhtml

Findings

Quantitative and qualitative findings from this mixed methods study are presented with seven key themes organized under the domains of facilitation, community engagement and program infra-structure and support summarised below as Table 3.

Table 3. Summary of key themes

Domain	Verbatim theme heading	Theme
Facilitation	They can watch if our words ring true	Community facilitation increased contextual validity
Community engagement	So, then we decided to take off our veils	Implementation affected and was affected by gender relations
	We talked about things I wanted to know	Responsiveness to group concerns increased participation
	I didn't plan to, but I ended up coming every week	Participation gathered momentum
Program infra-structure	We adjusted what we did depending on each group and each day	Training promoted a strong core and porous periphery
	I liked seeing Whatsapp pictures to know what they were doing in Agra too	Implementation was enhanced by technology
	My husband and I sat and talked about what went well after leading each week	Fidelity strengthened by critical reflection

Facilitation

"They can watch if our words ring true." – Community facilitators increased Parwarish contextual validity

Three coaches and 19 facilitators implemented Parwarish, and all (except one) were parents of an adolescent and lived in the target communities. Every facilitator reported that arguments with shouting or hitting occurred in their own household illustrating shared lived experience with participants. The 22 facilitators and coaches had a mean age of 39.9 years and 77% were from a Scheduled Caste or Tribe reflecting a similar demographic make-up to participants.

Parents and adolescents described a high level of trust with facilitators at two levels, firstly, through pre-existing relationships with the implementing teams; secondly, through facilitators who responded to their current needs and issues. One family described how the Parwarish facilitators supported them when their adolescent self-harmed and needed hospital care. A facilitator explains how their community base increased connection and trust below:

> *Because we live in this place anyone can see what work we do and how we do things all day long. If someone comes from outside, then people don't know how that person really lives but if we live in the same place, they can see if our words ring true*

For peer review only

For peer review only - http://bmjopen.bmj.com/site/about/guidelines.xhtml

in how we live. So, I think it is good to live in the same place as group members and also, I know the best way to explain things as I know about them and their lives.

SSI, Facilitator, Agra

Programme facilitation by community members also presented challenges: community facilitators described feeling scrutinised by their neighbours in their own parenting and communication ('they watch if our words ring true') and some struggled to observe professional work ethics, E.g., to avoid gossip and breaches of confidentiality. Facilitators also described less engagement from groups most proximate to their own homes as described below:

"Being a facilitator in my own village and among people of my caste is more challenging ( ) as they don't take my words seriously and that reduces impact. But in other castes and communities people respond to my words positively."

Facilitator, FGD, Robertsganj,

This mistrust was somewhat mitigated by the team taking pains to be punctual, reliable and prepared and increasing trust leading to increased participation as Parwarish progressed.

Community engagement

"So, then we decided to take off our veils" – gender relations affected and were affected by the intervention.

Fathers explained their higher attendance in Agra due to encouragement by male facilitators so that just 13 of 129 fathers were classified as programme dropouts (missing four consecutive sessions) while few fathers attended in Agra and Khunti. Mothers and adolescents in all the sites had a high level of attendance and completion of home activities and their Parwarish participation is summarised in Table 4.

Table 4. Summary of participation (attendance) in Parwarish

	**Mean sessions attended (out of 14)**	**Mean number of home catch up sessions delivered (for those who missed a session) (out of 13)**	**Mean number of home activities completed**
Mother	11.6	1.1	9.8
Father	5.8	3.3	4.8
Girls	11.2	1.0	10.5
Boys	11.1	1.1	10.2

The context of patriarchal gender relations which value men and disadvantage women impacted implementation in numerous ways. Implementers described difficulty in participation from men and boys who were less available due to income generation responsibilities. The lower rates of literacy for women also led to difficulties in identifying women as facilitators. Gender norms dictated women are commonly responsible for

For peer review only - http://bmjopen.bmj.com/site/about/guidelines.xhtml

nurturing aspects of parenting meaning many households 'assigned' attendance of sessions to mothers.

A positive aspect of the limited women working was that mothers and daughters were available for Parwarish sessions during the day except in tribal Jharkhand where coaches and facilitators described both men and women work full time meaning they both had limited discretionary time and perhaps more equal gender relations than main-stream North India.

Some resistance to traditional gender relations was evidenced through group participation. For example, in Robertsganj and Agra initially there was widespread practice of *pardah* (a gendered practice prevalent through North India dictating men and women related to each other should not sit together and women should hide their faces from older male family members). After several sessions, facilitators and several women decided to challenge status quo gender relations by sitting beside males, and unveiling their faces as described:

> *"I decided it would be improper for men and women to sit together and see each other so I also wore pardah for first two sessions. But then I said I also couldn't understand what other women with faces covered were saying. So then we all decided to take our veils off and we were all showing our eyes.( )After two or three sessions in our facilitator group we also discussed the practice of purdah and use of face coverings and we decided we could make our own new rules for this group."*
> **SSI, Female facilitator, Agra site**

"We talked about things I wanted to know" - responding to group concerns increased participation

The interactive format of Parwarish was identified as core to engagement, with sessions described as participatory and non-didactic. Facilitators described role-modelling the ideas, for example ensuring women and men participated in discussions while discussing the importance of family decision-making. Parents described sessions were responsive to their concerns as father describes below:

> *"I really liked coming to the group because we'd talk about things we wanted to know about and everyone gave me suggestions to solve all these problems my wife and I were having. Then after that we would also discuss about the parenting thing and that was interesting so then I decided to stay for all the sessions."*

Father, FGD, Agra

However, facilitators described challenges in using a non-didactic approach. They argued that eliciting responses made sessions lengthy and at times they omitted roleplays. Home activities (such as praising children or developing a family budget) helped keep families engaged. One mechanism of learning was that participants would teach the subject matter to others in their households, and even update remote family members by telephone as described below:

For peer review only - http://bmjopen.bmj.com/site/about/guidelines.xhtml

> *I really love the plays and stories in the Parwarish sessions and so once I brought along my sister Mira who was visiting from Delhi. Later Mira wanted to learn more so I would ring her after each session to tell her everything that happened. Then Mira's mother-in-law in Delhi had noticed Mira was being appreciative and praising her often which was a new thing, so her mother-in-law also wanted to learn about the session, so now Mira's whole household listens to the phone updates when I ring them each week.*

Mother, FGD, Agra

'I didn't mean to but I ended up coming every week' - participation gathered momentum

Participants described multiple positive feedback loops that increased participation, engagement and ultimately outcomes as Parwarish progressed. Parents described planning to attend just one session but then attending weekly as described below.

> *"I really liked the idea of learning more about how to parent my children but I felt like I didn't have time for this, so I thought I'll just go twice and stop. But after two sessions there were so many funny stories and examples from other parents having exactly the same problems as we have. So, then I decided that I would come every time.*

Father, FGD, Agra

Later in Parwarish men described taking missing their daily wage work to participate and meeting at times of day they initially said were inconvenient. Momentum was also evident when community members requested if they could join the next programme.

Program infra-structure

Parwarish had minimal physical infrastructure, as private homes and courtyards were used for group meetings however this also led to challenges such as private homes being too small for large groups which at times reduced participation and fidelity. However Parwarish used a substantial and formal implementation infrastructure through training, observed feedback and documentation.

"We adjusted what we did depending on each group and each day" - Training promoted a strong core and porous periphery

Facilitators described that the in-depth training and coaching provided a clear core intervention on which they made pragmatic micro-adaptations. Facilitators and coaches described applying Parwarish learnings to their own household leading to positive changes which increased their engagement and provided examples they used in group sessions. Micro-adaptations described by facilitators were in response to community requests or contextual and pragmatic such as changing the reducing group size from 15 to 10 families to fit with crowded urban settings or holding sessions in late evenings in villages where agricultural work dominated day-times.

For peer review only - http://bmjopen.bmj.com/site/about/guidelines.xhtml

"I liked looking at Whatsapp pictures to see what they were doing in Agra too" - Implementation enhanced by technology

Coaches and facilitators identified social media platform Whatsapp as central for communications, coordination, supervision and feedback in real time. Photos and text were shared many times daily to coordinate and facilitate problem-solving between implementers. For example, photos of groups in cramped small rooms led to the solution proposed to meet in a more spacious outdoor courtyard. Coaches and coordinating team members described how Whatsapp messages provided encouragement and increased facilitator activity. For example, the Agra team described felt support via Whatsapp when they had to run groups in a flooded courtyard so they persisted despite inconvenience. Messages also were used for coordination and data collection, such as to update baseline research forms filled, which motivated other sites, and increased compliance and participation for all teams.

"My husband and I sat and talked about what went well after leading each week" - Fidelity strengthened by critical reflection

There was 100% attendance in the Parwarish training by coaches and facilitators and attendance of facilitators and coaches at weekly meetings was over 90%. Fidelity scores filled by facilitators and through structured observation ranged from 3.5 to 4.1 of 5 (self-report) and 5.3- 5.5 (observed by coaches and researchers) suggestig fidelity to the intervention was fairly good for all sites. Observers noted that facilitators initially omitted module components when pressed for time and read roleplays aloud rather than acting them out. Review of the value of role plays after Module 5 led to improved fidelity. The key omissions in fidelity to documentation was in not filling registers to critically reflect on sessions (challenges, highlights) in the first 5-6 modules and this improved. Session duration was a mean of 1.5 hours (Khunti), 2.2 hours (Agra) and 2.8 hours (Robertsganj) with sessions in Khunti reportedly shorter due to the pressure of agricultural work in the tribal Jharkhand setting.

Parwarish implementation used formats and structures developed in PLH-Teen in South Africa to support critical reflection. For example, each facilitator filled a format after sessions describing highlights (what had gone well) as well as challenges (what didn't go well), and their responses to observations and these were discussed with their coach. A fortnightly meeting between coaches in the three sites, the coordination team and the PLH-Teens support team used Skype group conversations with a translator increased the quality of coordination, reporting and real-time responsiveness and reduced travel.

Facilitators described how the formational Parwarish training changed their own attitudes to gender equality in marriage relations and adult-child hierarchies. Coaches also described the fortnightly reports as improving their own group facilitation prompted them to help facilitators solve their own problems. An example of attitude change leading to behaviour change is described below:

> *"(In our weekly facilitator meeting) Sushila described how she had never asked her three sons to help in the kitchen, but the other facilitators described how their sons*

For peer review only - http://bmjopen.bmj.com/site/about/guidelines.xhtml

would make chappattis and all. ( ) So then Sushila told us how once getting late and so she had rung her son to ask him to wash dishes and he did that. She said it was a very positive experience."

Discussion

This process evaluation shows acceptability and relevance for the Parwarish intervention evidenced by high levels of attendance, participation and engagement by parents and adolescents in three deprived socio-economic settings in India.

Enrolment and attendance rates were significantly higher than in PLH-Teens implementations in South Africa, Philippines and Sudan (7, 23, 24). Factors likely to have increased attendance include context-relevant adaptations by the community-based facilitators and the pre-existing strong relationships and community development work of implementing teams. The novelty of a first-ever research project with daily Whatsapp group reporting across sites perhaps added a competitive component (25).

The results demonstrate Parwarish as feasibly implemented by lay community facilitators and coaches. This has positive implications for scaling: implementation by local people receiving modest remuneration in meeting spaces provided by the community makes this a relatively low-cost intervention (23). However, while the program is likely to be feasible in other disadvantaged settings in South Asia, the high level of commitment by facilitators (for example, being willing to conduct meetings at night in Jharkhand communities) may be harder to replicate. Implementers also underlined the value of structured, high-intensity program buttressing to increase fidelity and quality of Parwarish, which was also identified as central in PLH-Teens implementation (9, 20, 21) and other community-based group interventions in South Asia (26-28).

Implementers and facilitators described the India-specific adaptation of PLH-Teens as relevant and acceptable in two key ways: firstly, the curricular adaptations that were superficial (e.g. names of characters in role plays) as well as deeper changes, such as implementation by married couples and holding group meetings in the private homes / courtyards in Uttar Pradesh sites made Parwarish culturally appropriate and easy-to-engage-with in these settings(23, 24).

Secondly, relevance and acceptability were increased by trained community members facilitating groups, using their in-depth knowledge of the daily lives, culture and language (ie. context) of participants. Parwarish facilitators used local dialects and gave examples familiar to participants from shared common knowledge, adjusting logistics and roleplays to maximise participation. Implementers described the importance of trusting the innate contextual knowledge of facilitators (29) and the value of facilitators discussing their proposed adaptations to the programme with PLH-Teens coaches to ensure the core was followed but in locally acceptable ways. Facilitators described a range of continuous micro-adaptations, a feature key to successful interventions in other low- and middle-income

For peer review only - http://bmjopen.bmj.com/site/about/guidelines.xhtml

settings (26, 30, 31). At the same time, some of the changes such as meeting at night perhaps reduced visual learning (like role plays) as there was no electricity at night.

The effectiveness of Parwarish was enhanced through the program supports of coaching to increase facilitator competency, also a key theme in other PLH programmes (21, 23, 24). Strategies increasing intervention fidelity and quality included observation and feedback by coaches, weekly meetings, checklists and fortnightly ‘coach the coaches’ sessions. The fidelity to the programme was acceptable but required structured feedback problem solve barriers like meeting timing, and to include more challenging aspects like acting out role plays. The need for a balance between fidelity and ‘fit’ with the local context is widely described(21, 32, 33) and in these findings there were some compromises in fidelity to ‘fit’ the community needs.

This study was set in India, which ranks 140th out of 153 countries globally in the Global gender gap index and among the world’s bottom five countries in the health and survival category meaning there are large gender disparities that disadvantage women, (18)**.** Parwarish held transforming gender relations as core to both process and content and even implementing Parwarish with men and women together in a group confronted the predominant gender relations, an approach endorsed elsewhere in North India (34).

Gender relations influenced participation, for example decreasing attendance of fathers and sons, because of gender role expectations to generate income (7, 23, 24). There were also proportionately more girls among participating adolescents, perhaps reflecting their lower priority for school attendance and employment (18). Parwarish also enabled new attitudes and gender relations, for example, the Agra and Robertsganj group decision to not observe purdah which facilitators described as key to dialogue in the group meetings and leading to other outcomes where women described increased autonomy. This early outcome generated a sense of possibility of change to entrenched behaviours linked to harsh parenting and increased engagement of facilitators, mothers and young women, a feature important in gender transformative interventions (34).

Parwarish groups used face-to-face trainings and meetings, with facilitator manuals and monitoring formats both paper-based, yet implementation was strengthened through technological applications and mobile phones. In India there are over 1 billion users of mobile phones (35) and facilitators and coaches described three new ways technology enhanced their work: firstly in Whats app group communications between coaches and facilitators allowing pictures and video calls. Secondly for coordination with implementers in New Delhi and thirdly, in the use of Skype conversations for ‘coach the coaches’ sessions between field sites, New Delhi and South Africa as app described as familiar (widely used by peers), free and synchronous and easy to use. The benefits of Whatsapp to support problem solving and quality of community health programme implementation in low resourced settings has been described by others (25, 36) and further realised in the COVID-19 era (37, 38).

For peer review only - http://bmjopen.bmj.com/site/about/guidelines.xhtml

Methodological considerations

There are some important limitations to this study. First, researchers were involved in higher level implementation of this project (PS and PN) which could have led to social desirability bias in responses. A benefit of including implementers as researchers was that PS, KM and PN had an in-depth understanding of factors influencing implementation as well as contextual challenges. Second, the evaluation did not include a control group. Third, while researchers visited implementation sites several times, no researchers resided in intervention communities which will have reduced contextual understanding. There were important strengths in the study design: First, it reflected the 'real-world' allowing genuine assessment of feasibility and acceptability; Secondly, the implementation design where Parwarish was implemented by community members as facilitators, with a modest budget, in three diverse sites and among people from low-income communities which increased generalisability of findings to implementation by others with a limited budget working among groups living with disadvantage.

Conclusions

This evaluation demonstrates the Parwarish intervention as acceptable, relevant and feasible in three diverse settings of India: an urban slum and a rural agricultural town in North India and a remote tribal region of Central India. In a vast country which has seen almost no examples of programmes seeking to increase positive parenting and reduce family violence, this pilot study shows the implementation process was engaging, feasible and acceptable in communities, which paves the way for larger-scale implementation in other settings in India and South Asia.

Ethics statement

The Emmanuel Hospital Association Institutional Ethics committee, New Delhi approved this study (Protocol 191) in March 2019.

Contributor ship statement

KM conceived of the study, designed it with PS and IG and wrote the first draft, PN collected data with PS, and completed analysis with KM, PP and IG, and all authors contributed to multiple drafts of the paper.

Funding

This work was supported by the Swedish Research Council: 2017-05421.

Competing interests

All authors declare they had no competing interests in the implementation and evaluation of this intervention and study.

For peer review only - http://bmjopen.bmj.com/site/about/guidelines.xhtml

Data sharing

No additional data available

References

1. Patel R, Gupte SS, Srivastava S, Kumar P, Chauhan S, Govindu MD, et al. Experience of gender-based violence and its effect on depressive symptoms among Indian adolescent girls: Evidence from UDAYA survey. PLoS One. 2021;16(3):e0248396.
2. Deb S, Modak S. Prevalence of violence against children in families in Tripura and its relationship with socio-economic factors. J Inj Violence Res. 2010;2(1):5-18.
3. Daral S, Khokhar A, Pradhan S. Prevalence and Determinants of Child Maltreatment among School-Going Adolescent Girls in a Semi-Urban Area of Delhi, India. J Trop Pediatr. 2016;62(3):227-40.
4. Ellsberg M, Arango DJ, Morton M, Gennari F, Kiplesund S, Contreras M, et al. Prevention of violence against women and girls: what does the evidence say? The Lancet. 2015;385(9977):1555-66.
5. Norman RE, Byambaa M, De R, Butchart A, Scott J, Vos T. The long-term health consequences of child physical abuse, emotional abuse, and neglect: a systematic review and meta-analysis. PLoS Med. 2012;9(11):e1001349.
6. Pedersen GA, Smallegange E, Coetzee A, Hartog K, Turner J, Jordans MJ, et al. A systematic review of the evidence for family and parenting interventions in low-and middle-income countries: child and youth mental health outcomes. Journal of Child and Family Studies. 2019:1-20.
7. Cluver L, Meinck F, Yakubovich A, Doubt J, Redfern A, Ward C, et al. Reducing child abuse amongst adolescents in low- and middle-income countries: A pre-post trial in South Africa. BMC Public Health. 2016;16(1):567.
8. Cluver LD, Meinck F, Steinert JI, Shenderovich Y, Doubt J, Herrero Romero R, et al. Parenting for Lifelong Health: a pragmatic cluster randomised controlled trial of a non-commercialised parenting programme for adolescents and their families in South Africa. BMJ Glob Health. 2018;3(1):e000539.
9. Cluver L, Meinck F, Shenderovich Y, Ward CL, Romero RH, Redfern A, et al. A parenting programme to prevent abuse of adolescents in South Africa: study protocol for a randomised controlled trial. Trials. 2016;17(1):328.
10. Cluver L, Lachman J, Ward C, Gardner F, Petersen T, Meinck F, et al. Development of a parenting programme to prevent abuse of adolescents in South Africa: Findings from a pre-post pilot study. Research on Social Work Practice. 2016.
11. Lachman JM, Cluver LD, Boyes ME, Kuo C, Casale M. Positive parenting for positive parents: HIV/AIDS, poverty, caregiver depression, child behavior, and parenting in South Africa. AIDS Care. 2014;26(3):304-13.
12. Moore GF, Audrey S, Barker M, Bond L, Bonell C, Hardeman W, et al. Process evaluation of complex interventions: Medical Research Council guidance. BMJ : British Medical Journal. 2015;350:h1258.
13. Oakley A, Strange V, Stephenson J, Forrest S, Monteiro H. Evaluating processes: a case study of a randomized controlled trial of sex education. Evaluation. 2004;10(4):440-62.

For peer review only - http://bmjopen.bmj.com/site/about/guidelines.xhtml

14. Brand SL, Quinn C, Pearson M, Lennox C, Owens C, Kirkpatrick T, et al. Building programme theory to develop more adaptable and scalable complex interventions: Realist formative process evaluation prior to full trial. Evaluation. 2019;25(2):149-70.
15. Bowen DJ, Kreuter M, Spring B, Cofta-Woerpel L, Linnan L, Weiner D, et al. How we design feasibility studies. Am J Prev Med. 2009;36(5):452-7.
16. Colucci E, Hassan G. Prevention of domestic violence against women and children in low-income and middle-income countries. Current Opinion in Psychiatry. 2014;27(5):350-7.
17. Knerr W, Gardner F, Cluver L. Improving positive parenting skills and reducing harsh and abusive parenting in low-and middle-income countries: a systematic review. Prevention science. 2013;14(4):352-63.
18. World Economic Forum. Global Gender Gap report 2021. Geneva: World Economic Forum; 2021.
19. Government of India. Census of India 2011 New Delhi: Government of India; 2011. Contract No.: 29 September 2014.
20. Lachman JM, Cluver L, Ward CL, Hutchings J, Mlotshwa S, Wessels I, et al. Randomized controlled trial of a parenting program to reduce the risk of child maltreatment in South Africa. Child Abuse Negl. 2017;72:338-51.
21. Lachman JM, Kelly J, Cluver L, Ward CL, Hutchings J, Gardner F. Process evaluation of a parenting program for low-income families in South Africa. Research on Social Work Practice. 2018;28(2):188-202.
22. Braun V, Clarke V. Using thematic analysis in psychology. Qualitative research in psychology. 2006;3(2):77-101.
23. Janowski RK, Wessels I, Bojo S, Monday F, Maloney K, Achut V, et al. Transferability of evidence-based parenting programs to routine implementation in postconflict South Sudan. Research on Social Work Practice. 2020;30(8):858-69.
24. Alampay LP, Lachman JM, Landoy BV, Madrid BJ, Ward CL, Hutchings J, et al. Preventing Child Maltreatment in Low‐ and Middle-Income Countries: Parenting for Lifelong Health in the Philippines. Developmental science and sustainable development goals for children and youth: Springer; 2018. p. 277-93.
25. Henry JV, Winters N, Lakati A, Oliver M, Geniets A, Mbae SM, et al. Enhancing the Supervision of Community Health Workers With WhatsApp Mobile Messaging: Qualitative Findings From 2 Low-Resource Settings in Kenya. Global Health: Science and Practice. 2016;4(2):311-25.
26. Khan MN, Hamdani SU, Chiumento A, Dawson K, Bryant RA, Sijbrandij M, et al. Evaluating feasibility and acceptability of a group WHO trans-diagnostic intervention for women with common mental disorders in rural Pakistan: a cluster randomised controlled feasibility trial. Epidemiology and psychiatric sciences. 2017:1-11.
27. Leventhal KS, DeMaria LM, Gillham JE, Andrew G, Peabody J, Leventhal SM. A psychosocial resilience curriculum provides the "missing piece" to boost adolescent physical health: A randomized controlled trial of Girls First in India. Soc Sci Med. 2016;161:37-46.
28. Leventhal KS, Gillham J, DeMaria L, Andrew G, Peabody J, Leventhal S. Building psychosocial assets and wellbeing among adolescent girls: A randomized controlled trial. J Adolesc. 2015;45:284-95.
29. Shen J, Yang H, Cao H, Warfield C. The fidelity—Adaptation relationship in non-evidence-based programs and its implication for program evaluation. Evaluation. 2008;14(4):467-81.
30. Nakkash RT, Alaouie H, Haddad P, El Hajj T, Salem H, Mahfoud Z, et al. Process evaluation of a community-based mental health promotion intervention for refugee children. Health Educ Res. 2012;27(4):595-607.
31. Sharma S, Adetoro OO, Vidler M, Drebit S, Payne BA, Akeju DO, et al. A process evaluation plan for assessing a complex community-based maternal health intervention in Ogun State, Nigeria. BMC Health Serv Res. 2017;17(1):238.

For peer review only

For peer review only - http://bmjopen.bmj.com/site/about/guidelines.xhtml

32. Castro FG, Barrera M, Martinez CR. The cultural adaptation of prevention interventions: resolving tensions between fidelity and fit. Prevention Science. 2004;5(1):41-5.
33. Limbani F, Goudge J, Joshi R, Maar MA, Miranda JJ, Oldenburg B, et al. Process evaluation in the field: global learnings from seven implementation research hypertension projects in low-and middle-income countries. BMC Public Health. 2019;19(1):953.
34. Freudberg H, Contractor S, Das A, Kemp CG, Nevin PE, Phadiyal A, et al. Process and impact evaluation of a community gender equality intervention with young men in Rajasthan, India. Culture, health & sexuality. 2018:1-16.
35. Telecom regulatory authority of India. India's active mobile users reached 1,026.37 million. New Delhi: Telecom regulatory authority of India; 2019.
36. Perry H, Crigler L, Gergen J. Supervision of community health workers. In: Perry H, Crigler L, editors. Developing and strengthening community health worker programs at scale - aA reference guide and case studies for program managers and policymakers Baltimore, MD: MCHIP, USAID. Jhpiego Corporation; 2013.
37. Ajisegeri W, Odusanya O, Joshi R. Covid-19 outbreak situation in Nigeria and the need for effective engagement of community health workers for epidemic response. Global Biosecurity. 2020;1(4).
38. How WhatsApp became the tool of choice for WHO's COVID-19 messaging [press release]. 2020.

For peer review only

For peer review only - http://bmjopen.bmj.com/site/about/guidelines.xhtml

Supplementary files

Table 1 – Supplementary file

Table 3. Qualitative data collection overview

**Participants**	**Data collection**
Facilitators at each site	3 FGD with facilitators (n= 22 people)
Parents and adolescent participants in Parwarish	4 FGD with parents (n = 29) and 4 FGD with adolescents (n= 23)
Online coach session with 3 site coaches and implementation coordinator and researcher	Detailed notes of online session (total 11 pages of typed text)
Facilitators (community-based pairs)	6 individual interviews
Program implementers (Delhi and Agra based)	3 individual interviews
Coaches (located at each of the three field sites)	3 individual interviews
Observations of training, group facilitation in field and coaching sessions	Field notes (total 14 pages of typed text)
Fortnightly logbook documenting highlights and challenges on sessions filled by coaches and shared fortnightly	Fortnightly logbook shared as an email with PN and KM and South African CWB coach
Total number of people contributing to qualitative data	N=86

For peer review only

COREQ (COnsolidated criteria for REporting Qualitative research) Checklist

A checklist of items that should be included in reports of qualitative research. You must report the page number in your manuscript where you consider each of the items listed in this checklist. If you have not included this information, either revise your manuscript accordingly before submitting or note N/A.

Topic	Item No.	Guide Questions/Description	Reported on Page No.
**Domain 1: Research team and reflexivity**			
Personal characteristics			
Interviewer/facilitator	1	Which author/s conducted the interview or focus group?	
Credentials	2	What were the researcher's credentials? E.g. PhD, MD	
Occupation	3	What was their occupation at the time of the study?	
Gender	4	Was the researcher male or female?	
Experience and training	5	What experience or training did the researcher have?	
Relationship with participants			
Relationship established	6	Was a relationship established prior to study commencement?	
Participant knowledge of the interviewer	7	What did the participants know about the researcher? e.g. personal goals, reasons for doing the research	
Interviewer characteristics	8	What characteristics were reported about the inter viewer/facilitator? e.g. Bias, assumptions, reasons and interests in the research topic	
**Domain 2: Study design**			
Theoretical framework			
Methodological orientation and Theory	9	What methodological orientation was stated to underpin the study? e.g. grounded theory, discourse analysis, ethnography, phenomenology, content analysis	
Participant selection			
Sampling	10	How were participants selected? e.g. purposive, convenience, consecutive, snowball	
Method of approach	11	How were participants approached? e.g. face-to-face, telephone, mail, email	
Sample size	12	How many participants were in the study?	
Non-participation	13	How many people refused to participate or dropped out? Reasons?	
Setting			
Setting of data collection	14	Where was the data collected? e.g. home, clinic, workplace	
Presence of non-participants	15	Was anyone else present besides the participants and researchers?	
Description of sample	16	What are the important characteristics of the sample? e.g. demographic data, date	
Data collection			
Interview guide	17	Were questions, prompts, guides provided by the authors? Was it pilot tested?	
Repeat interviews	18	Were repeat inter views carried out? If yes, how many?	
Audio/visual recording	19	Did the research use audio or visual recording to collect the data?	
Field notes	20	Were field notes made during and/or after the inter view or focus group?	
Duration	21	What was the duration of the inter views or focus group?	
Data saturation	22	Was data saturation discussed?	
Transcripts returned	23	Were transcripts returned to participants for comment and/or	

For peer review only

Topic	Item No.	Guide Questions/Description	Reported on Page No.
		correction?	
**Domain 3: analysis and findings**			
Data analysis			
Number of data coders	24	How many data coders coded the data?	
Description of the coding tree	25	Did authors provide a description of the coding tree?	
Derivation of themes	26	Were themes identified in advance or derived from the data?	
Software	27	What software, if applicable, was used to manage the data?	
Participant checking	28	Did participants provide feedback on the findings?	
Reporting			
Quotations presented	29	Were participant quotations presented to illustrate the themes/findings? Was each quotation identified? e.g. participant number	
Data and findings consistent	30	Was there consistency between the data presented and the findings?	
Clarity of major themes	31	Were major themes clearly presented in the findings?	
Clarity of minor themes	32	Is there a description of diverse cases or discussion of minor themes?	

Developed from: Tong A, Sainsbury P, Craig J. Consolidated criteria for reporting qualitative research (COREQ): a 32-item checklist for interviews and focus groups. *International Journal for Quality in Health Care*. 2007. Volume 19, Number 6: pp. 349 – 357

Once you have completed this checklist, please save a copy and upload it as part of your submission. DO NOT include this checklist as part of the main manuscript document. It must be uploaded as a separate file.

For peer review only

BMJ Open

Is the Parwarish parenting intervention feasible and relevant for young people and parents in diverse settings in India? A mixed methods process evaluation

Journal:	*BMJ Open*
Manuscript ID	bmjopen-2021-054553.R1
Article Type:	Original research
Date Submitted by the Author:	15-Dec-2021
Complete List of Authors:	Mathias, Kaaren; Umea Universitet, Global health and epidemiology; Emmanuel Hospital Association, Community health and development<br>Nayak, Prabhudutt; Emmanuel Hospital Association, Chhatarpur Christian Hospital, Community health and development programme<br>Singh, Pratibha; Emmanuel Hospital Association<br>Pillai, Pooja; Emmanuel Hospital Association, Burans, Herbertpur Christian Hospital<br>Goicolea, I; Umea University, Global health and epidemiology
**Primary Subject Heading**:	Global health
Secondary Subject Heading:	Mental health, Public health, Qualitative research, Paediatrics
Keywords:	Public health < INFECTIOUS DISEASES, Non-accidental injury < PAEDIATRICS, Child & adolescent psychiatry < PSYCHIATRY

SCHOLARONE™
Manuscripts

I, the Submitting Author has the right to grant and does grant on behalf of all authors of the Work (as defined in the below author licence), an exclusive licence and/or a non-exclusive licence for contributions from authors who are: i) UK Crown employees; ii) where BMJ has agreed a CC-BY licence shall apply, and/or iii) in accordance with the terms applicable for US Federal Government officers or employees acting as part of their official duties; on a worldwide, perpetual, irrevocable, royalty-free basis to BMJ Publishing Group Ltd ("BMJ") its licensees and where the relevant Journal is co-owned by BMJ to the co-owners of the Journal, to publish the Work in this journal and any other BMJ products and to exploit all rights, as set out in our licence.

The Submitting Author accepts and understands that any supply made under these terms is made by BMJ to the Submitting Author unless you are acting as an employee on behalf of your employer or a postgraduate student of an affiliated institution which is paying any applicable article publishing charge ("APC") for Open Access articles. Where the Submitting Author wishes to make the Work available on an Open Access basis (and intends to pay the relevant APC), the terms of reuse of such Open Access shall be governed by a Creative Commons licence – details of these licences and which Creative Commons licence will apply to this Work are set out in our licence referred to above.

Other than as permitted in any relevant BMJ Author's Self Archiving Policies, I confirm this Work has not been accepted for publication elsewhere, is not being considered for publication elsewhere and does not duplicate material already published. I confirm all authors consent to publication of this Work and authorise the granting of this licence.

For peer review only

Title page

Is the Parwarish parenting intervention feasible and relevant for young people and parents in diverse settings in India? A mixed methods process evaluation

Kaaren Mathias, Prabhudutt Nayak, Pratibha Singh, Pooja Pillai, Isabel Goicolea

Corresponding Author

Kaaren Mathias

Institutional affiliation:

1. Dept of Global health and epidemiology, Universitet Umea, Sweden
2. Emmanuel Hospital Association, New Delhi, India
3. School of Health Sciences, University of Canterbury, Christchurch, New Zealand

169 Southampton St, Sydenham, Christchurch, New Zealand 8023

Email kaaren.mathias@umu.se

Ph +64 20 41497530

Co-authors

Prabhudutt Nayak

Emmanuel Hospital Association, Chhatarpur, India

Pratibha Singh

Emmanuel Hospital Association, New Delhi, India

Pooja Pillai

Herbertpur Christian Hospital, Herbertpur, India

Isabel Goicolea

Dept of Global health and epidemiology, Universitet Umea, Sweden

For peer review only - http://bmjopen.bmj.com/site/about/guidelines.xhtml

For peer review only

For peer review only - http://bmjopen.bmj.com/site/about/guidelines.xhtml

Keywords

India, parenting, family violence, process assessment, community

Word Count = 4459

For peer review only

For peer review only - http://bmjopen.bmj.com/site/about/guidelines.xhtml

Is the Parwarish parenting intervention feasible and relevant for young people and parents in diverse settings in India? A mixed methods process evaluation.

Kaaren Mathias, Prabhudutt Nayak, Pratibha Singh, Pooja Pillai, Isabel Goicolea

Abstract n=296 words

Objective

To assess the feasibility, acceptability and relevance of the Parwarish, a positive parenting intervention (adapted from PLH-Teens) in three diverse settings in India.

Design

This mixed methods study used the Medical Research Council framework for process evaluations of complex interventions.

Setting

This study was set in disadvantaged communities in urban Agra, rural Uttar Pradesh and tribal Jharkhand in India.

Participants

Data was collected from 86 facilitators, implementers, parents and teens who participated in the Parwarish intervention among 239 families.

Intervention

Couples from target communities facilitated groups of parents and teens over the 14 -module structured, interactive Parwarish intervention which focused on building communication, reducing harsh parenting and building family budgeting skills.

Outcome measures

We assessed relevance, acceptability and feasibility of the intervention using mixed methods. Qualitative data collected included semi-structured interviews and focus group discussions with implementers, facilitators, parents and young people that were transcribed, translated and thematically analysed to develop themes inherent in the data. Quantitative data which assessed attendance, fidelity to the intervention and facilitator training and coaching were analysed descriptively.

Results

Findings were grouped under the three domains of facilitation, community engagement and program support with the following seven themes: 1)community-based facilitators increased contextual validity of the intervention; 2) gender relations influenced Parwarish implementation, but were also influenced and transformed by Parwarish; 3) facilitator responsiveness to group concerns

For peer review only - http://bmjopen.bmj.com/site/about/guidelines.xhtml

increased participation; 4) participation gathered momentum; 5) Parwarish's strong core and porous periphery allowed adaptations to local contexts; 6) Technology that included Skype and Whats app enhanced implementation and 7) critical reflection with community trained coaches strengthened facilitation quality and program fidelity.

Conclusion

This study found Parwarish engaging, feasible and acceptable in three diverse, low-income communities, although constrained by patriarchal gender relations. It paves the way for larger-scale implementation in other South Asia settings.

Strengths and limitations of this study

- This study is set in three diverse settings providing greater generalisability
- Research conducted by programme implementers allows close-up understanding of the context
- There is a risk of social desirability bias in responses from participants due to research being conducted by some programme implementers.
- The 'real world' study design increases generalisability of the findings
- The 'real world' study design meant the intervention was adapted to different contexts, reducing fidelity to the programme.

Protocol

This study has no study protocol.

Acknowledgements

We thank the Jacob Gwal and Englina Ela Rani Tirkey (coach) with the Khunti team, Somesh Singh and Deepika Tikvah (coach) with the Agra team and Jyotsana Saha (coach) with the Robertsganj team for all their enthusiasm and diligent work implementing Parwarish and supporting with data collection for this study. We are also thankful for the support in adaptation of Parwarish to Jeet Bahadur, Prerana Singh, Amanda Raymond and the Experts by Experience group of mothers and others in the Community Health and Development programme of the Emmanuel Hospital Association.

For peer review only - http://bmjopen.bmj.com/site/about/guidelines.xhtml

Introduction

Young people in India experience high rates of physical, sexual and emotional abuse as well as neglect (1, 2) with a recent study estimating that 70% of adolescent girls in Delhi have experienced violence at some time in their lives (3). India has more adolescents than any other country, and the millions affected by violence risk current and life-long problems with physical and mental health, well-being, education and employment (1, 4, 5). India ranked 140th globally in the latest Global Gender gap report (6) and gender inequality is a key driver of gender based and family violence in India(1).

Positive parenting interventions can potentially reduce violence in two key ways: firstly by promoting parenting strategies which reduce physically or emotionally violent actions, and secondly by reducing violent or anti-social behaviour among children(7). Parenting in India has not been studied widely and a large systematic review examining interventions to reduce harsh parenting or promote positive parenting in low and middle income countries could not identify any studies located in India (7). Studies examining parenting of adolescents in middle-class urban settings identify parenting is often authoritarian, extends until early adulthood, with a focus on educational attainment, and with a high level of monitoring of the social behaviour of their teenagers (particularly young women)(8-10). However, we could find no interventional studies promoting positive parenting among disadvantaged adolescents in India.

There is growing evidence that interventions focusing on positive parenting can reduce abuse of young people and children (11-13). Further, the most effective programmes are participatory, address underlying risk factors like gender relations and support reduced non-violent behaviours, greater communication and shared decision making among family members. Yet globally, 10% or fewer of adolescents can access violence prevention programs.(4, 14).

Parenting for Lifelong Health is a positive parenting group intervention developed in South Africa for parents and adolescents, that builds on social learning and parent management training principles seeking reduce all types of violence in families (15). Other publications detail PLH-Teens' development, theory, and effectiveness (12-16). PLH-Teens was adapted for the Indian setting by the[1] Emmanuel Hospital Association (EHA) working in consultation with a group of mothers of adolescents (experts by experience) from disadvantaged communities in Dehradun, Uttarakhand. Together they reviewed both the superficial structures (language, role plays) as well as cultural content and named the adapted intervention Parwarish (meaning 'nurturing' in Hindi). This paper reports a process evaluation of Parwarish in three diverse communities in Central and North India.

Process evaluations are valuable to assess the fidelity and quality of implementation, to clarify causal mechanisms and identify contextual factors associated with variation in outcomes and to build interventions that are more generalisable and ultimately scalable (17-19). Examining feasibility assesses whether an intervention is relevant, acceptable and how it can be adapted contextually (20). There is very limited research documenting implementation of family violence prevention

[1] EHA is a non-profit organisation working across North and North-East India with charitable hospitals, community health and development programmes.

For peer review only - http://bmjopen.bmj.com/site/about/guidelines.xhtml

programmes, particularly in community and low- and middle-income settings (4, 5, 7, 21). Many describe the urgent need for studies that examine context and implementation processes in order to better develop interventions that reduce violence in communities, particularly in low- and middle-income settings (4, 5, 7, 11, 19, 21).

This study aimed to examine whether the Parwarish parenting intervention was feasible, relevant and acceptable for parents and adolescents and community facilitators living in three diverse and disadvantaged communities in Central and Northern India, in order to establish the utility of this intervention for scaled implementation across other South Asian settings. .

Methods

Setting of the intervention

This study examined Parwarish implementation in three diverse settings: In Agra, a large city in Uttar Pradesh, in Robertsganj, a small town in the rural Sonbhadra district, Uttar Pradesh and tribal Khunti, Jharkhand, a rural and remote setting. The context of the intervention is detailed further in the results section.

Intervention

Intervention content

Parwarish seeks to reduce harsh parenting and violence within families through new attitudes and skill building between parents and adolescents. Content includes spending quality time together, communicating positively, managing strong emotions like anger and finding safe support when needed as well as problem solving, conflict resolution and finance skills, including how to manage a household budget and uses role-plays, activity-based learning and home rehearsal and activities building on social learning (15, 16, 22, 23). Further detail of the 14 modules of the Parwarish curriculum is provided in Supplementary materials, Table 2.

Implementation

Parwarish was implemented by the EHA Community health and development programme teams based in Agra, Robertsganj (UP) and Khunti (Jharkhand). The budget for both implementation and evaluation was limited and therefore we conducted the programme using in-kind support by Emmanuel Hospital Association existing resources and team members. A coach was appointed and trained for each location and took responsibility for recruiting facilitators as well as training and coaching facilitators. Separately, a project officer at each location was responsible for research components of the project and supported baseline and endline data collection as well as monitoring and evaluation of Parwarish sessions with other EHA community coordinators in the team.

Implementation of Parwarish groups was conducted by pairs of community facilitators with the following criterion for facilitation selection:

For peer review only - http://bmjopen.bmj.com/site/about/guidelines.xhtml

- parents of adolescents who were resident in the target community.
- represent an equal mix of genders willing to work as a pair in facilitation (over half of facilitators worked as a married couple).
- trusted and accepted as a leader by the community.
- effective communicators.
- had obtained at least 10th class pass and were fluent in the local dialect or language.

Parwarish training and coaching

Trainers from PLH-Teens South Africa facilitated a 10-day course for Parwarish facilitators, and a 3-day training for coaches (total n=25 participants). The facilitators then led the 14 Parwarish modules with groups of parents and teens with meetings of 1.5 to two hours and encouraged participants to complete the weekly activity to try at home E.g., family eats dinner together. Home visits with those who missed a session review the topic for the week. In each site, a local 'coach' supported facilitators with weekly meetings reflecting on their facilitation while a fortnightly coach-the-coaches meeting was led online with someone from PLH-Teens South Africa.

Recruitment of target communities and participants

EHA community health and development team members with support from community volunteers recruited families in communities where they had been running community health and development programmes in the previous five or more years, by informing them about Parwarish and inviting participation. They held small community meetings informing about the proposed programme and invited any families who met the inclusion criteria and keen to participate to give their names and details to a team member. Inclusion criteria were:

- at least one adolescent (aged 12 – 18 years) in the household
- permanent resident in the target community
- at least one parent/ carer and one adolescent available for weekly sessions.

A total of 239 of 245 families invited agreed to join the programme (97.6%). Based on team capacity there were 129 families recruited from Agra, 60 families from Robertsganj and 50 from tribal Khunti.

Data collection

In-depth interviews and focus group discussions (FGDs) were collected face-to-face in the community by KM, PS and PN in Hindi with 86 purposively sampled facilitators, parents and young people and continued until data saturation was reached. They lasted between 30 and 70 minutes and were recorded, translated and transcribed into English. Interview guides were developed by authors and piloted in the community. They asked about implementation processes, acceptability and feasibility as well as focus group discussions (FGD) with parents, adolescents and facilitators. Additional field notes and free text was recorded in coach and facilitator documents. Further data was collected during meetings with coaches which were conducted online, in the form of written notes taken during

For peer review only - http://bmjopen.bmj.com/site/about/guidelines.xhtml

meetings by a researcher not involved in meeting facilitation or participation (KM). Supplementary Table 1 provides an overview of the qualitative data collection.

Measures of fidelity

Coaches also filled a paper register at each site on the following measures:

- Facilitation and fidelity: 14 of 14 sessions self-reported by facilitators using a 5-point checklist where a score of 1 indicated Poor quality and a score of 5 indicated High quality. The following components of the intervention were assessed with this scale: physical exercise, emotional check in, main teaching topic, role play and completion of home activity.
- Facilitation and fidelity: 4 of 14 sessions for each Parwarish group were observed and measured by the coach, EHA project manager or a researcher who had all been trained to fill using the same criteria. Fidelity assessed the five aspects above and two additional aspects, namely: facilitator engagement, and completion of registers.
- Attendance of facilitators and coaches at trainings and coaching: attendance register.
- Attendance of parents and adolescents: attendance register
- Completion of assigned home activities: register.
- Home visits completed by facilitators: register.

Conceptual framework and analysis

This mixed methods study was analysed with a merging of two evaluation frameworks, namely process and feasibility evaluation, as we sought to understand how the intervention was delivered, and also whether it was feasible and acceptable. We followed the Medical Research Council framework for process evaluation of complex interventions examining how the intervention was delivered, and the emerging adaptations of the intervention related to context and actors (17). We also assessed feasibility which addresses facets of implementation that include practicality, pragmatic and implementation processes used to deliver Parwarish programme to participants, adaptations to the Parwarish intervention required to increase engagement, understanding and relevance to diverse communities. Additionally, we examined acceptability and demand for Parwarish by asking participants whether they were comfortable with the group implementation processes and programme content (20).

Qualitative interviews and written documents were read and re-read and analysed with thematic analysis developed by Braun and Clarke (24). Four transcripts were inductively coded by PN and KM and these codes were reviewed to develop a coding framework, paying attention to implementation processes, feasibility and barriers and enablers of the intervention. Remaining transcripts were then coded with this framework by KM and PN, who collaboratively developed categories and then themes, looking for patterns inherent in

For peer review only - http://bmjopen.bmj.com/site/about/guidelines.xhtml

the data. Quantitative data was analysed descriptively and tabulated however given geographically dispersed field sites data was not member checked with parents and children in communities.

Patient and public involvement statement

Parents were involved in this study in three key ways. Firstly, parents were the impetus for this Parwarish intervention, having requested EHA team members in Dehradun, Agra and Khunti to support them to build their skills in raising young people. Secondly, parents were part of the process of adaptation of the intervention. A group of parents who resided in informal communities in Dehradun were consulted as "Experts by experience" in reviewing and suggesting contextually relevant adaptations of Parwarish. Thirdly, parents from intervention target communities were identified and trained as facilitators of this intervention and thus were also involved in recruiting participants, and collecting data linked to implementation required by facilitators such as the highlights and lowlights register. Parents in communities will also be involved in sharing findings at dissemination meetings planned after COVID-19 restrictions are lifted later in 2021 using community meetings and reviewing a plain-language Hindi summary of study findings.

Stance and ethics

This study was conducted by participant researchers who had all lived in India for at least 24 years and spoke fluent Hindi. KM, PS, PP (women) and PN (man) were employed by the implementers Emmanuel Hospital Association. KM (PhD) facilitated adaptation of PLH-Teens to Parwarish and led meetings with experts by experience. PS and PN (MPH and MSW) supported Parwarish implementation at a high level. PP (M.Med Sci) and IG (PhD) are women who work in India and Sweden respectively. Ethics approval was granted by the Institutional Ethics committee of EHA in Delhi for this study (Protocol 191) in February 2019.

Findings

Findings are presented under the broad headings of Context and Implementation proposed in the process evaluation of complex interventions guide of the MRC (17). We present broad features of the diverse contexts of each Parwarish implementation site. Evaluation of implementation presents quantitative and qualitative data summarising fidelity, dose and reach, followed by thematic analysis of the qualitative data presented under the domains of facilitation, community engagement and program infra-structure. Mechanisms of impact are described within the qualitative thematic analysis.

For peer review only - http://bmjopen.bmj.com/site/about/guidelines.xhtml

Context

Parwarish was implemented by teams from the Community development and Health programme (CHDP) of the Emmanuel Hospital Association in North India. The diverse study sites are summarised with a demographic profile of their three districts compared with national Indian data in Table 1. While all three sites report high proportions of young people and high rates of gender-based violence, the Jharkhand site is predominantly tribal and rural, while Agra is urban with a marked asymmetry in the sex ratio. Only half of women in the UP rural site of Sohbhadra are literate and the majority live rurally. Gender inequality is a key contextual factor that influenced implementation across all sites, and this is evidenced in Table 1. Asymmetries in the sex ratios of live births (fewer females) as well as the lower literacy rates of women underline the dominant patriarchy which is further discussed in qualitative findings of implementation.

Table 1. Socio-demographic profile of the three districts where Parwarish was implemented, with reference data for India nationally (25)

Indicator	National - India	Agra City, Uttar Pradesh	Sonbhadra district, Uttar Pradesh	Khunti district, Jharkhand
Total population (million people)	1,200	4.41	1.86	0.53
% population rural	72.2	54.2	83.1	91.5
% population under 18 years (State)	34.9	33.7	33.7	33.5
% population Scheduled castes/Scheduled Tribes	25.2	22.6	14.9	77
Sex ratio (female to 1000 males)	940	868	918	997
Literacy (% literate female)	65.5	61.2	52.1	53.7
Literacy (% literate male)	82.1	80.6	74.9	74.1
Prevalence of gender-based violence (% at State level)	40	47	47	30 – 50

The context is further described by considering the socio-demographic profile of community participants profiled in Table 2. This demonstrates participants were highly disadvantaged and where over 80% of participants were from Scheduled Tribes or Scheduled castes, which are classifications assigned at birth and utilised by the Indian Government to indicate people groups who are structurally disadvantaged. Further indications of the social and economic disadvantage of participants are the fact that 55% of fathers and 75% of mothers hadn't completed primary schooling.

Table 2. Socio-demographic profile of Parwarish participants

(N=239 families)	Agra (%)	Robertsganj (%)	Khunti (%)	Parent

For peer review only - http://bmjopen.bmj.com/site/about/guidelines.xhtml

	Adolescents/household			Mother	Father
**Female**	68 (52.7)	36 (60.0)	32 (64.0)	-	
**Age in years (Mean)**	13.3	13.3	14.0	36.8	40.2
**Caste (Indian census classification) :**					
Scheduled caste or tribes	110 (85.3)	51 (85)	50 (100)	199(83%)	
Other backward castes	19 (14.7)	9 (15)	-	37 (15%)	
General	-	-	-	3 (1.3%)	
**Religion:**					
Muslim	11 (8.5)	3 (5)	-	14 (6%)	
Hindu	117 (90.7)	57 (95)	3 (6)	167 (70%)	
Christian	-	-	21 (42)	29 (12%)	
Other religion	-	-	26 (52)	29 (12%)	
**Education:**					
Never been to school	31 (24.0)	-	-	135 (56%)	83 (35%)
Class 5 completed	46 (35.7)	32 (53.3)	23 (46)	48 (20%)	48 (20%)
Class 10 completed	44 (34.2)	48 (46.6)	20 (40)	43 (18%)	83 (35%)
Class 12 completed	8 (6.2)	-	3 (6)	2 (1%)	7 (3%)

Fidelity, dose and reach

Fidelity

Fidelity scores filled by facilitators and through each aspect of structured observation ranged from 70.0 – 82.0% (in a self-report five-item measure) and 75.7 – 78.6% (in a seven-item observed measure filled by coaches and researchers) suggesting fidelity to the intervention was moderate to good for all sites. Observers noted that facilitators initially omitted module components when pressed for time and read roleplays aloud rather than acting them out. Review of the value of role plays after Module 5 led to improved fidelity in delivery of this component of the intervention. The key omissions in fidelity to documentation was in failing to write detailed comments in registers to critically reflect on sessions (challenges, highlights) in the first 5-6 modules. After review and discussion with facilitators this improved. Session duration was a mean of 1.5 hours (Khunti), 2.2 hours (Agra) and 2.8 hours (Robertsganj) with sessions in Khunti reportedly shorter due to the pressure of agricultural work in the tribal Jharkhand setting.

Dose and reach

There was 100% attendance in the Parwarish training by coaches and facilitators. Participant evaluations of Parwarish training were very positive, noting that there was opportunity to rehearse also put into practice components of the intervention. For example, training facilitators took turns leading the morning ‘emotional check-in’ and each also worked in pairs to develop their own household budget, a key output of later modules. Attendance of facilitators and coaches at weekly coaching meetings was over 90%. Attendance and engagement of participants in Parwarish is summarised below in Table 3.

For peer review only - http://bmjopen.bmj.com/site/about/guidelines.xhtml

Participation was higher for women and adolescents than for men. Fathers explained their higher attendance in Agra was due to encouragement by male facilitators so that just 13 of 129 fathers were classified as programme dropouts (missing four consecutive sessions) while few fathers attended in Agra and Khunti, reflecting dominant gender relations where men are expected to generate income.

For peer review only

For peer review only - http://bmjopen.bmj.com/site/about/guidelines.xhtml

Table 3. Summary of participation (attendance) in Parwarish

	Mean sessions attended (out of 14)	Mean number of home catch up sessions delivered (for those who missed a session) (out of 13)	Mean number of home activities completed
Mother	11.6	1.1	9.8
Father	5.8	3.3	4.8
Girls	11.2	1.0	10.5
Boys	11.1	1.1	10.2

Implementation

The seven themes that emerged are presented under three key domains linked to implementation of the Parwarish intervention and are summarised in Table 4. A verbatim theme heading is provided to give greater texture to themes and is further illustrated with verbatim quotes taken from interviews and focus group discussions.

Table 4. Summary of key themes

Domain	Theme	Verbatim theme heading
Facilitation	Community facilitation increased contextual validity of Parwarish	They can watch if our words ring true
Community engagement	Implementation affected and was affected by gender relations	So, then we decided to take off our veils
	Responsiveness increased participation	We talked about things I wanted to know
	Participation gathered momentum during implementation	I didn't plan to, but I ended up coming every week
Program infra-structure	Training promoted a strong core for implementation	We adjusted what we did depending on each group and each day
	Implementation was enhanced by technology	I liked seeing Whatsapp pictures to know what they were doing in Agra too
	Fidelity was strengthened by critical reflection	My husband and I sat and talked about what went well after facilitating each week

Facilitation

"They can watch if our words ring true." – Community facilitators increased Parwarish contextual validity

Three coaches and 19 facilitators implemented Parwarish, and all (except one) were parents of an adolescent and lived in the target communities. Every facilitator reported that

For peer review only - http://bmjopen.bmj.com/site/about/guidelines.xhtml

arguments with shouting or hitting occurred in their own household illustrating shared lived experience with participants. The 22 facilitators and coaches had a mean age of 39.9 years and 77% were from a Scheduled Caste or Tribe reflecting a similar demographic make-up to participants.

Parents and adolescents described a high level of trust with facilitators at two levels, firstly, through pre-existing relationships with the implementing teams; secondly, through facilitators who responded to their current needs and issues. One family described how the Parwarish facilitators supported them when their adolescent self-harmed and needed hospital care. A facilitator explains how their community base increased connection and trust below:

> *Because we live in this place anyone can see what work we do and how we do things all day long. If someone comes from outside, then people don't know how that person really lives but if we live in the same place, they can see if our words ring true in how we live. So, I think it is good to live in the same place as group members and also, I know the best way to explain things as I know about them and their lives.*

SSI, Facilitator, Agra

Programme facilitation by community members also presented challenges: community facilitators described feeling scrutinised by their neighbours in their own parenting and communication ('they watch to see if our words ring true') and some struggled to observe professional work ethics, E.g., to avoid gossip and breaches of confidentiality. Facilitators also described less engagement from groups most proximate to their own homes as described below:

> *"Being a facilitator in my own village and among people of my caste is more challenging ( ) as they don't take my words seriously and that reduces impact. But in other castes and communities people respond to my words positively."*

Facilitator, FGD, Robertsganj,

This mistrust was somewhat mitigated by the team taking pains to be punctual, reliable and prepared and increasing trust leading to increased participation as Parwarish progressed.

Community engagement

"So, then we decided to take off our veils" – gender relations affected and were affected by the intervention.

The context of patriarchal gender relations which value men and disadvantage women impacted implementation in numerous ways. Implementers described reduced participation from men and boys who were less available due to income generation responsibilities. The lower rates of literacy for women also led to difficulties in identifying women as facilitators.

For peer review only - http://bmjopen.bmj.com/site/about/guidelines.xhtml

Gender norms dictated women are commonly responsible for nurturing aspects of parenting meaning many households 'assigned' attendance of sessions to mothers.

A positive aspect of the gender norms which limited women from doing paid work in Agra was that mothers and daughters were available for Parwarish sessions during the day although in tribal Jharkhand both men and women work full time meaning reflecting tribal communities practice perhaps more equal gender relations than main-stream North India.

The sessions were facilitated in a way that sought to overcome typical age and gender related restrictions that might limit participation by young women, a key mechanism to support outcomes. Facilitators described how they sought participation from all with no 'right' or 'wrong' responses. A young woman described how this gave her courage to speak up:

> *Before this Parwarish course I would never give my opinion in front of adults I didn't know. But Anupriya (woman facilitator) would ask me to say what I thought and she said that there was no right or wrong answer so I could just give my opinion. Then I shared my opinion nearly every session and also I started to share my opinion at school.*

Young woman, Agra, FGD

Some resistance to traditional gender relations was evidenced through group participation which facilitators thought was triggered by both role modelling by facilitators as well as the process of equal valuing of participation by group members. For example, in Robertsganj and Agra initially there was widespread practice of *pardah* (a gendered practice prevalent through North India dictating men and women related to each other should not sit together and women should hide their faces from older male family members). After several sessions, facilitators and several women decided to challenge status quo gender relations by sitting beside males, and unveiling their faces as described:

> *"I decided it would be improper for men and women to sit together and see each other so I also wore pardah for first two sessions. But then I said I also couldn't understand what other women with faces covered were saying. So then we all decided to take our veils off and we were all showing our eyes.( )After two or three sessions in our facilitator group we also discussed the practice of purdah and use of face coverings and we decided we could make our own new rules for this group."*

SSI, Female facilitator, Agra site

For peer review only - http://bmjopen.bmj.com/site/about/guidelines.xhtml

"We talked about things I wanted to know" - responding to group concerns increased participation

The interactive format of Parwarish was identified as core to engagement, with sessions described as participatory and non-didactic. Facilitators described role-modelling the ideas as a key mechanism supporting outcomes, for example ensuring women and men participated in discussions while discussing the importance of family decision-making. Parents described sessions were responsive to their concerns as father describes below:

> *"I really liked coming to the group because we'd talk about things we wanted to know about and everyone gave me suggestions to solve all these problems my wife and I were having. Then after that we would also discuss about the parenting thing and that was interesting so then I decided to stay for all the sessions."*

Father, FGD, Agra

Role plays also provided participants with 'positive' and 'negative' examples of how to engage in family negotiations and communications about relevant issues to this community, and were identified as a key mechanism to provoke thought and attitude change. A young women described how the role play approach helped her discuss her hopes for study with her parents:

> *I was really wanting to talk about how my parents had said I should stop attending school, but I didn't know how. Then in one of the role plays about Nagma she discusses with her mother what she really wants to do. I really loved that role play and then I could discuss with my mother about my hope that I want to do more school study*

Teenage girl, FGD, Agra

However, facilitators also described challenges in using a non-didactic approach. They argued that eliciting responses made sessions lengthy and at times they then felt there was not time to complete roleplays. However, the home activities (such as praising children or developing a family budget) helped keep families engaged. One mechanism of learning was that young people described that they would revise and teach the subject matter to their siblings in their households. A mother also described how she would re-teach the weekly topic to remote family members by telephone as described below:

> *I really love the plays and stories in the Parwarish sessions and so once I brought along my sister Mira who was visiting from Delhi. Later Mira wanted to learn more so I would ring her after each session to tell her everything that happened. Then Mira's mother-in-law in Delhi had noticed Mira was being appreciative and praising her often which was a new thing, so her mother-in-law also wanted to learn about the*

For peer review only - http://bmjopen.bmj.com/site/about/guidelines.xhtml

session, so now Mira's whole household listens to the phone updates when I ring them each week.

Mother, FGD, Agra

'I didn't mean to but I ended up coming every week' - participation gathered momentum

Participants described multiple positive feedback loops that increased participation, engagement and ultimately outcomes as Parwarish progressed. Positive factors described included enjoyment of the group role plays and games, a sense of growing friendship with other participants, hearing others stories of similar struggles and appreciating new ideas in content of sessions. Parents described planning to attend just one session but then attending weekly as described below.

> *"I really liked the idea of learning more about how to parent my children but I felt like I didn't have time for this, so I thought I'll just go twice and stop. But after two sessions there were so many funny stories and examples from other parents having exactly the same problems as we have. So, then I decided that I would come every time.*

Father, FGD, Agra

Later in Parwarish men described taking missing their daily wage work to participate and meeting at times of day they initially said were inconvenient. Momentum was also evident when adolescents, younger children and other ineligible community members requested if they could join the next programme.

Program infra-structure

Parwarish had minimal physical infrastructure, as private homes and courtyards were used for group meetings however this also led to challenges such as homes were often too small for large groups which at times reduced participation and fidelity. However fidelity was documented and maintained largely through training, observed feedback and documentation.

"We adjusted what we did depending on each group and each day" - Training promoted a strong core

Facilitators described that the in-depth training and coaching provided a clear core intervention on which they made pragmatic micro-adaptations as a more porous periphery to the intervention. Facilitators and coaches described applying Parwarish learnings to their own household leading to positive changes which increased their engagement and provided examples they used in group sessions. Micro-adaptations described by facilitators were in response to community requests or contextual and pragmatic such as changing the reducing

For peer review only - http://bmjopen.bmj.com/site/about/guidelines.xhtml

group size from 15 to 10 families to fit with crowded urban settings or holding sessions in late evenings in villages where agricultural work dominated day-times.

"I liked looking at Whatsapp pictures to see what they were doing in Agra too" - Implementation enhanced by technology

Coaches and facilitators identified social media platform Whatsapp as central for communications, coordination, supervision and feedback in real time. Photos and text were shared many times daily to coordinate and facilitate problem-solving between implementers. For example, photos of groups in cramped small rooms led to the solution proposed to meet in a more spacious outdoor courtyard. Coaches and coordinating team members described how Whatsapp messages provided encouragement and they identified a sense of competition and ability to showcase work as a mechanism that promoted them to work harder as facilitators. For example, the Agra team described how they felt support from others via Whatsapp when they had to run groups in a flooded courtyard so they persisted despite inconvenience. Messages also were used for coordination and data collection, such as to update baseline research forms filled, which motivated other sites, and increased compliance and participation for all teams.

"My husband and I sat and talked about what went well after leading each week" - Fidelity strengthened by critical reflection

Parwarish implementation used formats and structures developed in PLH-Teen in South Africa as a key mechanism to trigger critical reflection. For example, each facilitator filled a format after sessions describing highlights (what had gone well) as well as challenges (what didn't go well), and their responses to observations and these were discussed with their coach. A fortnightly meeting between coaches in the three sites, the coordination team and the PLH-Teens support team used Skype group conversations with a translator increased the quality of coordination, reporting and real-time responsiveness and reduced travel.

Facilitators described how the formational Parwarish training changed their own attitudes to gender equality in marriage relations and adult-child (age) hierarchies. Coaches also described the fortnightly reports as improving their own group facilitation prompted them to help facilitators solve their own problems. An example of attitude change leading to behaviour change is described below:

> *"(In our weekly facilitator meeting) Sushila described how she had never asked her three sons to help in the kitchen, but the other facilitators described how their sons would make chapatis and all. ( ) So then Sushila told us how once getting late and so she had rung her son to ask him to wash dishes and he did that. She said it was a very positive experience."*

For peer review only - http://bmjopen.bmj.com/site/about/guidelines.xhtml

Discussion

This process evaluation shows acceptability and relevance for the Parwarish intervention evidenced by high levels of attendance, participation and engagement by parents and adolescents in three deprived socio-economic settings in India.

Enrolment and attendance rates for fathers and mothers in Parwarish were significantly higher than in PLH-Teens implementations in South Africa, Philippines and Sudan (12, 26, 27). Factors likely to have increased attendance include context-relevant adaptations by the community-based facilitators and the pre-existing strong relationships and community development work of implementing teams. The novelty of a first-ever research project with daily Whatsapp group reporting across sites perhaps added a competitive component that promoted greater activity among facilitators (28).

The results demonstrate Parwarish as feasibly implemented by lay community facilitators and coaches. This has positive implications for scaling across South Asia and beyond: implementation by local people receiving modest remuneration in meeting spaces provided by the community makes this a relatively low-cost intervention (26). However, while the program is likely to be feasible and acceptable in other disadvantaged settings in South Asia, the high level of commitment by facilitators (for example, being willing to conduct meetings at night in Jharkhand communities) may be harder to replicate. Implementers also underlined the value of structured, high-intensity program buttressing to increase fidelity and quality of Parwarish, which was also identified as central in PLH-Teens implementation (14, 22, 23) and other community-based group interventions in South Asia (29-31).

Implementers and facilitators described the India-specific adaptation of PLH-Teens as relevant and acceptable in two key ways: firstly, the curricular adaptations that were superficial (e.g. names of characters in role plays) were appropriate and felt recognisable, while deeper changes, such as implementation by married couples and holding group meetings in the private homes / courtyards in Uttar Pradesh sites made Parwarish feasible, culturally appropriate and easy-to-engage-with in these settings(26, 27).

Secondly, relevance and acceptability were increased by deployment of trained community members facilitating groups, using their in-depth knowledge of the daily lives, culture and language (ie. context) of participants. Parwarish facilitators used local dialects in facilitation and gave examples familiar to participants from shared common knowledge, adjusting logistics and roleplays in each setting to maximise participation. Implementers described the importance of trusting the innate contextual knowledge of facilitators (32) and the value of facilitators discussing their proposed adaptations to the programme with PLH-Teens coaches to ensure the core was followed but in locally acceptable ways. Facilitators described a range of continuous micro-adaptations, a feature key to successful interventions in other low- and middle-income settings (29, 33, 34). At the same time, some of the

For peer review only - http://bmjopen.bmj.com/site/about/guidelines.xhtml

changes such as meeting at night perhaps reduced visual learning (like role plays) as there was no electricity at night.

The effectiveness of Parwarish was enhanced through the program supports of coaching to increase facilitator competency, also a key theme in other PLH programmes (23, 26, 27). Strategies increasing intervention fidelity and quality included observation and feedback by coaches, weekly meetings, checklists and fortnightly 'coach the coaches' sessions. The fidelity to the programme was acceptable but required structured feedback to solved problems such as meeting timing, and needed attentive discussion to address more challenging aspects like acting out role plays which were omitted due to discomfort in initial modules. The need for a balance between fidelity and 'fit' with the local context is widely described(23, 35, 36) and in these findings there were some compromises in fidelity to 'fit' the community needs.

While participation of fathers in Parwarish was greater than has occurred in other PLH-Teens settings, men's attendance was still significantly less than participation by women. Men's participation in any family linked intervention is critical as they typically control financial decisions and dominate family decision-making in many North Indian households(37, 38). Strategies to do this increase participation of men include including men as facilitators, ensuring meetings are held at times when men can attend, and developing momentum for men's participation by engaging with men who are 'positive deviants'(37). India is among the world's bottom five countries in the health and survival category of the Global gender gap index meaning there are large gender disparities that disadvantage women and value men, (6)**.** Parwarish held transforming gender relations as core to both process and content and implementation of Parwarish with men and women together in a group confronted the predominant gender relations, an approach endorsed elsewhere in North India (37).

Gender relations influenced participation, for example decreasing attendance of fathers and sons, because of gender role expectations to generate income (12, 26, 27). There were also proportionately more girls among participating adolescents, perhaps reflecting their lower priority for school attendance and employment (6). Parwarish also enabled new attitudes and gender relations. For example, the Agra and Robertsganj groups' decision to not observe purdah was as key to dialogue in the group meetings and leading to other outcomes where women described increased autonomy. This early outcome generated a sense of possibility of change to entrenched behaviours linked to harsh parenting and increased engagement of facilitators, mothers and young women, a feature important in gender transformative interventions (37). Addressing deeply embedded gender relations of patriarchy prevalent across South Asia has no quick fix solution, and further work on gender transformative interventions is urgently needed in communities, schools, and national policies across India to bring greater gender equality to benefit both women and men(37).

For peer review only - http://bmjopen.bmj.com/site/about/guidelines.xhtml

Parwarish groups used face-to-face trainings and meetings, with facilitator manuals and monitoring formats both paper-based, yet implementation was strengthened through technological applications and mobile phones. In India there are over 1 billion users of mobile phones (39) and facilitators and coaches described three new ways technology enhanced their work: firstly in Whatsapp group communications between coaches and facilitators allowing pictures and video calls. Secondly for coordination with implementers in New Delhi and thirdly, in the use of Skype conversations for 'coach the coaches' sessions between field sites, New Delhi and South Africa as app described as familiar (widely used by peers), free and synchronous and easy to use. The benefits of Whatsapp to support problem solving and quality of community health programme implementation in low resourced settings has been described by others (28, 40) and further realised in the COVID-19 era (41, 42).

Methodological considerations

There are some important limitations to this study. First, researchers were involved in higher level implementation of this project (PS and PN) which could have led to social desirability bias in responses. A benefit of including implementers as researchers was that PS, KM and PN had an in-depth understanding of factors influencing implementation as well as contextual challenges. Second, the evaluation did not include a control group and was evaluated immediately after implementation limiting knowledge about sustainability of changes, and if possible a future study would examine outcomes one year after completion of the intervention to assure whether changes are enduring.. Third, while researchers visited implementation sites several times, no researchers resided in intervention communities which will have reduced their contextual understanding which may have reduced understanding of the relevance and acceptability of the implementation process. There were important strengths in the study design: First, it reflected the 'real-world' allowing iterative micro-adaptations of the intervention to make it optimally relevant and acceptable in the community. Secondly, implementation by community members as facilitators, with groups housed in communities using local homes to host groups and a modest budget in three diverse sites provides a model for implementation that can be generalised for implementation by others working among people from low-income communities increases in low income communities across South Asia.

Conclusions

This evaluation demonstrates the Parwarish intervention as acceptable, relevant and feasible in three diverse settings of India: an urban slum and a rural agricultural town in North India and a remote tribal region of Central India. In a vast country which has seen almost no examples of programmes seeking to increase positive parenting and reduce family violence, this pilot study shows the implementation process was engaging, feasible

For peer review only - http://bmjopen.bmj.com/site/about/guidelines.xhtml

and acceptable in communities, which paves the way for larger-scale implementation in other settings in India and South Asia.

Ethics statement

The Emmanuel Hospital Association Institutional Ethics committee, New Delhi approved this study (Protocol 191) in March 2019.

Contributor ship statement

KM conceived of the study, designed it with PS and IG and wrote the first draft, PN collected data with PS, and completed analysis with KM, PP and IG, and all authors contributed to multiple drafts of the paper.

Funding

This work was supported by the Swedish Research Council: 2017-05421.

Competing interests

All authors declare they had no competing interests in the implementation and evaluation of this intervention and study.

Data sharing

No additional data available

References

1. Patel R, Gupte SS, Srivastava S, Kumar P, Chauhan S, Govindu MD, et al. Experience of gender-based violence and its effect on depressive symptoms among Indian adolescent girls: Evidence from UDAYA survey. PLoS One. 2021;16(3):e0248396.
2. Deb S, Modak S. Prevalence of violence against children in families in Tripura and its relationship with socio-economic factors. J Inj Violence Res. 2010;2(1):5-18.
3. Daral S, Khokhar A, Pradhan S. Prevalence and Determinants of Child Maltreatment among School-Going Adolescent Girls in a Semi-Urban Area of Delhi, India. J Trop Pediatr. 2016;62(3):227-40.
4. Ellsberg M, Arango DJ, Morton M, Gennari F, Kiplesund S, Contreras M, et al. Prevention of violence against women and girls: what does the evidence say? The Lancet. 2015;385(9977):1555-66.

For peer review only - http://bmjopen.bmj.com/site/about/guidelines.xhtml

5. Norman RE, Byambaa M, De R, Butchart A, Scott J, Vos T. The long-term health consequences of child physical abuse, emotional abuse, and neglect: a systematic review and meta-analysis. PLoS Med. 2012;9(11):e1001349.
6. World Economic Forum. Global Gender Gap report 2021. Geneva: World Economic Forum; 2021.
7. Knerr W, Gardner F, Cluver L. Improving positive parenting skills and reducing harsh and abusive parenting in low-and middle-income countries: a systematic review. Prevention science. 2013;14(4):352-63.
8. de Wit EE, Chakranarayan C, Bunders-Aelen JF, Regeer BJ. Learning about parenting together: a programme to support parents with inter-generational concerns in Pune, India. Contemp Fam Ther. 2018;40(1):68-83.
9. Isaac R, Annie I, Prashanth H. Parenting in India. Parenting across cultures: Springer; 2014. p. 39-45.
10. Brar P, Dworkin J, Jang J. Association of parenting with sexual attitudes and behaviors of college students in India. Sexuality & Culture. 2018;22(4):1037-53.
11. Pedersen GA, Smallegange E, Coetzee A, Hartog K, Turner J, Jordans MJ, et al. A systematic review of the evidence for family and parenting interventions in low-and middle-income countries: child and youth mental health outcomes. Journal of Child and Family Studies. 2019:1-20.
12. Cluver L, Meinck F, Yakubovich A, Doubt J, Redfern A, Ward C, et al. Reducing child abuse amongst adolescents in low- and middle-income countries: A pre-post trial in South Africa. BMC Public Health. 2016;16(1):567.
13. Cluver LD, Meinck F, Steinert JI, Shenderovich Y, Doubt J, Herrero Romero R, et al. Parenting for Lifelong Health: a pragmatic cluster randomised controlled trial of a non-commercialised parenting programme for adolescents and their families in South Africa. BMJ Glob Health. 2018;3(1):e000539.
14. Cluver L, Meinck F, Shenderovich Y, Ward CL, Romero RH, Redfern A, et al. A parenting programme to prevent abuse of adolescents in South Africa: study protocol for a randomised controlled trial. Trials. 2016;17(1):328.
15. Cluver L, Lachman J, Ward C, Gardner F, Petersen T, Meinck F, et al. Development of a parenting programme to prevent abuse of adolescents in South Africa: Findings from a pre-post pilot study. Research on Social Work Practice. 2016.
16. Lachman JM, Cluver LD, Boyes ME, Kuo C, Casale M. Positive parenting for positive parents: HIV/AIDS, poverty, caregiver depression, child behavior, and parenting in South Africa. AIDS Care. 2014;26(3):304-13.
17. Moore GF, Audrey S, Barker M, Bond L, Bonell C, Hardeman W, et al. Process evaluation of complex interventions: Medical Research Council guidance. BMJ : British Medical Journal. 2015;350:h1258.
18. Oakley A, Strange V, Stephenson J, Forrest S, Monteiro H. Evaluating processes: a case study of a randomized controlled trial of sex education. Evaluation. 2004;10(4):440-62.
19. Brand SL, Quinn C, Pearson M, Lennox C, Owens C, Kirkpatrick T, et al. Building programme theory to develop more adaptable and scalable complex interventions: Realist formative process evaluation prior to full trial. Evaluation. 2019;25(2):149-70.
20. Bowen DJ, Kreuter M, Spring B, Cofta-Woerpel L, Linnan L, Weiner D, et al. How we design feasibility studies. Am J Prev Med. 2009;36(5):452-7.
21. Colucci E, Hassan G. Prevention of domestic violence against women and children in low-income and middle-income countries. Current Opinion in Psychiatry. 2014;27(5):350-7.
22. Lachman JM, Cluver L, Ward CL, Hutchings J, Mlotshwa S, Wessels I, et al. Randomized controlled trial of a parenting program to reduce the risk of child maltreatment in South Africa. Child Abuse Negl. 2017;72:338-51.

For peer review only

23. Lachman JM, Kelly J, Cluver L, Ward CL, Hutchings J, Gardner F. Process evaluation of a parenting program for low-income families in South Africa. Research on Social Work Practice. 2018;28(2):188-202.
24. Braun V, Clarke V. Using thematic analysis in psychology. Qualitative research in psychology. 2006;3(2):77-101.
25. Government of India. Census of India 2011 New Delhi: Government of India; 2011. Contract No.: 29 September 2014.
26. Janowski RK, Wessels I, Bojo S, Monday F, Maloney K, Achut V, et al. Transferability of evidence-based parenting programs to routine implementation in postconflict South Sudan. Research on Social Work Practice. 2020;30(8):858-69.
27. Alampay LP, Lachman JM, Landoy BV, Madrid BJ, Ward CL, Hutchings J, et al. Preventing Child Maltreatment in Low‐ and Middle-Income Countries: Parenting for Lifelong Health in the Philippines. Developmental science and sustainable development goals for children and youth: Springer; 2018. p. 277-93.
28. Henry JV, Winters N, Lakati A, Oliver M, Geniets A, Mbae SM, et al. Enhancing the Supervision of Community Health Workers With WhatsApp Mobile Messaging: Qualitative Findings From 2 Low-Resource Settings in Kenya. Global Health: Science and Practice. 2016;4(2):311-25.
29. Khan MN, Hamdani SU, Chiumento A, Dawson K, Bryant RA, Sijbrandij M, et al. Evaluating feasibility and acceptability of a group WHO trans-diagnostic intervention for women with common mental disorders in rural Pakistan: a cluster randomised controlled feasibility trial. Epidemiology and psychiatric sciences. 2017:1-11.
30. Leventhal KS, DeMaria LM, Gillham JE, Andrew G, Peabody J, Leventhal SM. A psychosocial resilience curriculum provides the “missing piece” to boost adolescent physical health: A randomized controlled trial of Girls First in India. Soc Sci Med. 2016;161:37-46.
31. Leventhal KS, Gillham J, DeMaria L, Andrew G, Peabody J, Leventhal S. Building psychosocial assets and wellbeing among adolescent girls: A randomized controlled trial. J Adolesc. 2015;45:284-95.
32. Shen J, Yang H, Cao H, Warfield C. The fidelity—Adaptation relationship in non-evidence-based programs and its implication for program evaluation. Evaluation. 2008;14(4):467-81.
33. Nakkash RT, Alaouie H, Haddad P, El Hajj T, Salem H, Mahfoud Z, et al. Process evaluation of a community-based mental health promotion intervention for refugee children. Health Educ Res. 2012;27(4):595-607.
34. Sharma S, Adetoro OO, Vidler M, Drebit S, Payne BA, Akeju DO, et al. A process evaluation plan for assessing a complex community-based maternal health intervention in Ogun State, Nigeria. BMC Health Serv Res. 2017;17(1):238.
35. Castro FG, Barrera M, Martinez CR. The cultural adaptation of prevention interventions: resolving tensions between fidelity and fit. Prevention Science. 2004;5(1):41-5.
36. Limbani F, Goudge J, Joshi R, Maar MA, Miranda JJ, Oldenburg B, et al. Process evaluation in the field: global learnings from seven implementation research hypertension projects in low-and middle-income countries. BMC Public Health. 2019;19(1):953.
37. Freudberg H, Contractor S, Das A, Kemp CG, Nevin PE, Phadiyal A, et al. Process and impact evaluation of a community gender equality intervention with young men in Rajasthan, India. Culture, health & sexuality. 2018:1-16.
38. Theobald S, Morgan R, Hawkins K, Ssali S, George A, Molyneux S. The importance of gender analysis in research for health systems strengthening. Health Policy Plan. 2017;32(suppl_5):v1-v3.
39. Telecom regulatory authority of India. India’s active mobile users reached 1,026.37 million. New Delhi: Telecom regulatory authority of India; 2019.
40. Perry H, Crigler L, Gergen J. Supervision of community health workers. In: Perry H, Crigler L, editors. Developing and strengthening community health worker programs at scale - aA reference guide and case studies for program managers and policymakers Baltimore, MD: MCHIP, USAID. Jhpiego Corporation; 2013.

For peer review only - http://bmjopen.bmj.com/site/about/guidelines.xhtml

41. Ajisegeri W, Odusanya O, Joshi R. Covid-19 outbreak situation in Nigeria and the need for effective engagement of community health workers for epidemic response. Global Biosecurity. 2020;1(4).
42. How WhatsApp became the tool of choice for WHO's COVID-19 messaging [press release]. 2020.

For peer review only

For peer review only - http://bmjopen.bmj.com/site/about/guidelines.xhtml

Supplementary files

Supplementary file 1: Overview of Parwarish curriculum content

Session	Key content
1	Introducing the programme and setting participant goals
2	The value of spending time together to build a positive parent – child relationship
3	Why praise and appreciation build connection – and practicing praise
4	Identifying and talking about emotions in the family
5	Managing anger without hurting others
6	Identifying problems and following steps for problem solving
7/8	The value of saving money and how to make a budget
9	Addressing problems without conflict (2 sessions)
10	Developing family rules and routines to reduce conflict and have fun
11	Ways to save money and make a family savings plan
12	Identifying risks and ways to keep safe in the community
13	How to manage in crisis – safeguards and strategies
14	Getting help and support beyond your household: widening the circle

Supplementary file 2: Qualitative data collection overview

**Participants**	**Data collection**
Facilitators at each site	3 FGD with facilitators (n= 22 people)
Parents and adolescent participants in Parwarish	4 FGD with parents (n = 29) and 4 FGD with adolescents (n= 23)
Online coach session with 3 site coaches and implementation coordinator and researcher	Detailed notes of online session (total 11 pages of typed text)
Facilitators (community-based pairs)	6 individual interviews
Program implementers (Delhi and Agra based)	3 individual interviews
Coaches (located at each of the three field sites)	3 individual interviews
Observations of training, group facilitation in field and coaching sessions	Field notes (total 14 pages of typed text)
Fortnightly logbook documenting highlights and challenges on sessions filled by coaches and shared fortnightly	Fortnightly logbook shared as an email with PN and KM and South African CWB coach
Total number of people contributing to qualitative data	N=86

For peer review only

For peer review only

COREQ (COnsolidated criteria for REporting Qualitative research) Checklist

A checklist of items that should be included in reports of qualitative research. You must report the page number in your manuscript where you consider each of the items listed in this checklist. If you have not included this information, either revise your manuscript accordingly before submitting or note N/A.

Topic	Item No.	Guide Questions/Description	Reported on Page No.
**Domain 1: Research team and reflexivity**			
Personal characteristics			
Interviewer/facilitator	1	Which author/s conducted the interview or focus group?	
Credentials	2	What were the researcher's credentials? E.g. PhD, MD	
Occupation	3	What was their occupation at the time of the study?	
Gender	4	Was the researcher male or female?	
Experience and training	5	What experience or training did the researcher have?	
Relationship with participants			
Relationship established	6	Was a relationship established prior to study commencement?	
Participant knowledge of the interviewer	7	What did the participants know about the researcher? e.g. personal goals, reasons for doing the research	
Interviewer characteristics	8	What characteristics were reported about the inter viewer/facilitator? e.g. Bias, assumptions, reasons and interests in the research topic	
**Domain 2: Study design**			
Theoretical framework			
Methodological orientation and Theory	9	What methodological orientation was stated to underpin the study? e.g. grounded theory, discourse analysis, ethnography, phenomenology, content analysis	
Participant selection			
Sampling	10	How were participants selected? e.g. purposive, convenience, consecutive, snowball	
Method of approach	11	How were participants approached? e.g. face-to-face, telephone, mail, email	
Sample size	12	How many participants were in the study?	
Non-participation	13	How many people refused to participate or dropped out? Reasons?	
Setting			
Setting of data collection	14	Where was the data collected? e.g. home, clinic, workplace	
Presence of non-participants	15	Was anyone else present besides the participants and researchers?	
Description of sample	16	What are the important characteristics of the sample? e.g. demographic data, date	
Data collection			
Interview guide	17	Were questions, prompts, guides provided by the authors? Was it pilot tested?	
Repeat interviews	18	Were repeat inter views carried out? If yes, how many?	
Audio/visual recording	19	Did the research use audio or visual recording to collect the data?	
Field notes	20	Were field notes made during and/or after the inter view or focus group?	
Duration	21	What was the duration of the inter views or focus group?	
Data saturation	22	Was data saturation discussed?	
Transcripts returned	23	Were transcripts returned to participants for comment and/or	

For peer review only

Topic	Item No.	Guide Questions/Description	Reported on Page No.
		correction?	
**Domain 3: analysis and findings**			
Data analysis			
Number of data coders	24	How many data coders coded the data?	
Description of the coding tree	25	Did authors provide a description of the coding tree?	
Derivation of themes	26	Were themes identified in advance or derived from the data?	
Software	27	What software, if applicable, was used to manage the data?	
Participant checking	28	Did participants provide feedback on the findings?	
Reporting			
Quotations presented	29	Were participant quotations presented to illustrate the themes/findings? Was each quotation identified? e.g. participant number	
Data and findings consistent	30	Was there consistency between the data presented and the findings?	
Clarity of major themes	31	Were major themes clearly presented in the findings?	
Clarity of minor themes	32	Is there a description of diverse cases or discussion of minor themes?	

Developed from: Tong A, Sainsbury P, Craig J. Consolidated criteria for reporting qualitative research (COREQ): a 32-item checklist for interviews and focus groups. *International Journal for Quality in Health Care*. 2007. Volume 19, Number 6: pp. 349 – 357

Once you have completed this checklist, please save a copy and upload it as part of your submission. DO NOT include this checklist as part of the main manuscript document. It must be uploaded as a separate file.

For peer review only

BMJ Open

Is the Parwarish parenting intervention feasible and relevant for young people and parents in diverse settings in India? A mixed methods process evaluation

Journal:	*BMJ Open*
Manuscript ID	bmjopen-2021-054553.R2
Article Type:	Original research
Date Submitted by the Author:	03-Feb-2022
Complete List of Authors:	Mathias, Kaaren; Umea Universitet, Global health and epidemiology; Emmanuel Hospital Association, Community health and development<br>Nayak, Prabhudutt; Emmanuel Hospital Association, Chhatarpur Christian Hospital, Community health and development programme<br>Singh, Pratibha; Emmanuel Hospital Association<br>Pillai, Pooja; Emmanuel Hospital Association, Burans, Herbertpur Christian Hospital<br>Goicolea, I; Umea University, Global health and epidemiology
**Primary Subject Heading**:	Global health
Secondary Subject Heading:	Mental health, Public health, Qualitative research, Paediatrics
Keywords:	Public health < INFECTIOUS DISEASES, Non-accidental injury < PAEDIATRICS, Child & adolescent psychiatry < PSYCHIATRY

SCHOLARONE™
Manuscripts

I, the Submitting Author has the right to grant and does grant on behalf of all authors of the Work (as defined in the below author licence), an exclusive licence and/or a non-exclusive licence for contributions from authors who are: i) UK Crown employees; ii) where BMJ has agreed a CC-BY licence shall apply, and/or iii) in accordance with the terms applicable for US Federal Government officers or employees acting as part of their official duties; on a worldwide, perpetual, irrevocable, royalty-free basis to BMJ Publishing Group Ltd ("BMJ") its licensees and where the relevant Journal is co-owned by BMJ to the co-owners of the Journal, to publish the Work in this journal and any other BMJ products and to exploit all rights, as set out in our licence.

The Submitting Author accepts and understands that any supply made under these terms is made by BMJ to the Submitting Author unless you are acting as an employee on behalf of your employer or a postgraduate student of an affiliated institution which is paying any applicable article publishing charge ("APC") for Open Access articles. Where the Submitting Author wishes to make the Work available on an Open Access basis (and intends to pay the relevant APC), the terms of reuse of such Open Access shall be governed by a Creative Commons licence – details of these licences and which Creative Commons licence will apply to this Work are set out in our licence referred to above.

Other than as permitted in any relevant BMJ Author's Self Archiving Policies, I confirm this Work has not been accepted for publication elsewhere, is not being considered for publication elsewhere and does not duplicate material already published. I confirm all authors consent to publication of this Work and authorise the granting of this licence.

For peer review only

Title page

Is the Parwarish parenting intervention feasible and relevant for young people and parents in diverse settings in India? A mixed methods process evaluation

Kaaren Mathias, Prabhudutt Nayak, Pratibha Singh, Pooja Pillai, Isabel Goicolea

Corresponding Author

Kaaren Mathias

Institutional affiliation:

1. Dept of Global health and epidemiology, Universitet Umea, Sweden
2. Emmanuel Hospital Association, New Delhi, India
3. School of Health Sciences, University of Canterbury, Christchurch, New Zealand

169 Southampton St, Sydenham, Christchurch, New Zealand 8023

Email kaaren.mathias@umu.se

Ph +64 20 41497530

Co-authors

Prabhudutt Nayak

Emmanuel Hospital Association, Chhatarpur, India

Pratibha Singh

Emmanuel Hospital Association, New Delhi, India

Pooja Pillai

Herbertpur Christian Hospital, Herbertpur, India

I Goicolea

Dept of Global health and epidemiology, Universitet Umea, Sweden

For peer review only

For peer review only

For peer review only - http://bmjopen.bmj.com/site/about/guidelines.xhtml

Keywords

India, parenting, family violence, process assessment, community

Word Count = 4459

For peer review only

For peer review only - http://bmjopen.bmj.com/site/about/guidelines.xhtml

Is the Parwarish parenting intervention feasible and relevant for young people and parents in diverse settings in India? A mixed methods process evaluation.

Kaaren Mathias, Prabhudutt Nayak, Pratibha Singh, Pooja Pillai, Isabel Goicolea

Abstract n=296 words

Objective

To assess the feasibility, acceptability and relevance of the Parwarish, a positive parenting intervention (adapted from PLH-Teens) in three diverse settings in India.

Design

This mixed methods study used the Medical Research Council framework for process evaluations of complex interventions.

Setting

This study was set in disadvantaged communities in urban Agra, rural Uttar Pradesh and tribal Jharkhand in India.

Participants

Data was collected from 86 facilitators, implementers, parents and teens who participated in the Parwarish intervention among 239 families.

Intervention

Couples from target communities facilitated groups of parents and teens over the 14 -module structured, interactive Parwarish intervention which focused on building communication, reducing harsh parenting and building family budgeting skills.

Outcome measures

We assessed relevance, acceptability and feasibility of the intervention using mixed methods. Qualitative data collected included semi-structured interviews and focus group discussions with implementers, facilitators, parents and young people that were transcribed, translated and thematically analysed to develop themes inherent in the data. Quantitative data which assessed attendance, fidelity to the intervention and facilitator training and coaching were analysed descriptively.

Results

Findings were grouped under the three domains of facilitation, community engagement and program support with the following seven themes: 1)community-based facilitators increased contextual validity of the intervention; 2) gender relations influenced Parwarish implementation, but were also influenced and transformed by Parwarish; 3) facilitator responsiveness to group concerns

For peer review only - http://bmjopen.bmj.com/site/about/guidelines.xhtml

increased participation; 4) participation gathered momentum; 5) Parwarish's strong core and porous periphery allowed adaptations to local contexts; 6) Technology that included Skype and Whats app enhanced implementation and 7) critical reflection with community trained coaches strengthened facilitation quality and program fidelity.

Conclusion

This study found Parwarish engaging, feasible and acceptable in three diverse, low-income communities, although constrained by patriarchal gender relations. It paves the way for larger-scale implementation in other South Asian settings.

Strengths and limitations of this study

- This study is set in three diverse settings providing greater generalisability
- Research conducted by programme implementers allows close-up understanding of the context
- There is a risk of social desirability bias in responses from participants due to research being conducted by some programme implementers.
- The 'real world' study design increases generalisability of the findings
- The 'real world' study design meant the intervention was adapted to different contexts, reducing fidelity to the programme.

Protocol

This study has no study protocol.

Acknowledgements

We thank the Jacob Gwal and Englina Ela Rani Tirkey (coach) with the Khunti team, Somesh Singh and Deepika Tikvah (coach) with the Agra team and Jyotsana Saha (coach) with the Robertsganj team for all their enthusiasm and diligent work implementing Parwarish and supporting with data collection for this study. We are also thankful for the support in adaptation of Parwarish to Jeet Bahadur, Prerana Singh, Amanda Raymond and the Experts by Experience group of mothers and others in the Community Health and Development programme of the Emmanuel Hospital Association.

For peer review only - http://bmjopen.bmj.com/site/about/guidelines.xhtml

Introduction

Young people in India experience high rates of physical, sexual and emotional abuse as well as neglect (1, 2) with a recent study estimating that 70% of adolescent girls in Delhi have experienced violence at some time in their lives (3). India has more adolescents than any other country, and the millions affected by violence risk current and life-long problems with physical and mental health, well-being, education, and employment (1, 4, 5). India ranked 140th globally in the latest Global Gender gap report (6) and gender inequality is a key driver of gender based and family violence in India(1).

Positive parenting interventions can potentially reduce violence in two key ways: firstly by promoting parenting strategies which reduce physically or emotionally violent actions, and secondly by reducing violent or anti-social behaviour among children(7). Parenting in India has not been studied widely and a large systematic review examining interventions to reduce harsh parenting or promote positive parenting in low and middle income countries could not identify any studies located in India (7). Studies examining parenting of adolescents in middle-class urban settings identify parenting is often authoritarian, extends until early adulthood, with a focus on educational attainment, and typically includes a high level of monitoring of the social behaviour of their teenagers (particularly young women)(8-10). However, we could find no interventional studies promoting positive parenting among disadvantaged adolescents in India.

There is growing evidence that interventions focusing on positive parenting can reduce abuse of young people and children (11-13). Further, the most effective programmes are participatory, address underlying risk factors like gender relations and support reduced non-violent behaviours, greater communication and shared decision making among family members. Yet globally, 10% or fewer of adolescents can access violence prevention programs.(4, 14).

Parenting for Lifelong Health is a positive parenting group intervention developed in South Africa for parents and adolescents, that builds on social learning and parent management training principles seeking reduce all types of violence in families (15). Other publications detail PLH-Teens' development, theory, and effectiveness (12-16). PLH-Teens was adapted for the Indian setting by the Emmanuel Hospital Association (EHA) [1] working in consultation with a group of mothers of adolescents (experts by experience) from disadvantaged communities in Dehradun, Uttarakhand. Together they reviewed both the superficial structures (language, role plays) as well as cultural content and named the adapted intervention Parwarish (meaning 'nurturing' in Hindi). This paper reports a process evaluation of Parwarish in three diverse communities in Central and North India.

Process evaluations are valuable to assess the fidelity and quality of implementation, to clarify causal mechanisms and identify contextual factors associated with variation in outcomes and to build interventions that are more generalisable and ultimately scalable (17-19). Examining feasibility assesses whether an intervention is relevant, acceptable, and how it can be adapted contextually (20). There is very limited research documenting implementation of family violence prevention

[1] EHA is a non-profit organisation working across North and North-East India with charitable hospitals, community health and development programmes.

For peer review only - http://bmjopen.bmj.com/site/about/guidelines.xhtml

programmes, particularly in community and low- and middle-income settings (4, 5, 7, 21). Many describe the urgent need for studies that examine context and implementation processes in order to better develop interventions that reduce violence in communities, particularly in low- and middle-income settings (4, 5, 7, 11, 19, 21).

This study aimed to examine whether the Parwarish parenting intervention was feasible, relevant and acceptable for parents and adolescents and community facilitators living in three diverse and disadvantaged communities in Central and Northern India, in order to establish the utility of this intervention for scaled implementation across other South Asian settings.

Methods

Setting of the intervention

This study examined Parwarish implementation in Agra, a large city in Uttar Pradesh, in Robertsganj, a small town in the rural Sonbhadra district, Uttar Pradesh and among indigenous people in Khunti, Jharkhand, a rural and remote setting. The context of the intervention is detailed further in the results section.

Intervention

Intervention content

Parwarish seeks to reduce harsh parenting and violence within families through new attitudes and skill building between parents and adolescents. Module content includes spending quality time together, communicating positively, managing strong emotions like anger and finding safe support when needed as well as problem solving, conflict resolution and finance skills, including how to manage a household budget and uses role-plays, activity-based learning and home rehearsal and activities building on social learning (15, 16, 22, 23). Further detail of the 14 modules of the Parwarish curriculum is provided in Supplementary materials, Table 1.

Implementation

Parwarish was implemented by the EHA Community health and development programme teams based in Agra, Robertsganj (UP) and Khunti (Jharkhand). The budget for both implementation and evaluation was limited and therefore we conducted the programme using in-kind support by Emmanuel Hospital Association existing resources and team members. A coach was appointed and trained for each location and took responsibility for recruiting facilitators as well as training and coaching facilitators. Separately, a project officer at each location was responsible for research components of the project and supported baseline and endline data collection as well as monitoring and evaluation of Parwarish sessions with other EHA community coordinators in the team.

Implementation of Parwarish groups was conducted by pairs of community facilitators with the following criterion for facilitation selection:

For peer review only - http://bmjopen.bmj.com/site/about/guidelines.xhtml

- parents of adolescents who were resident in the target community.
- represent an equal mix of genders willing to work as a pair in facilitation (over half of facilitators worked as a married couple).
- trusted and accepted as a leader by the community.
- effective communicators.
- had obtained at least 10th class pass and were fluent in the local dialect or language.

Parwarish training and coaching

Trainers from PLH-Teens South Africa facilitated a 10-day course for Parwarish facilitators, and a 3-day training for coaches (total n=25 participants). The facilitators then led the 14 Parwarish modules with groups of parents and teens with meetings of 1.5 to two hours and encouraged participants to complete the weekly activity to try at home E.g., family eats dinner together. Home visits with those who missed a session review the topic for the week. In each site, a local 'coach' supported facilitators with weekly meetings reflecting on their facilitation while a fortnightly coach-the-coaches meeting was led online with someone from PLH-Teens South Africa.

Recruitment of target communities and participants

EHA community health and development team members with support from community volunteers recruited families in communities where they had been running community health and development programmes in the previous five or more years, by informing them about Parwarish and inviting participation. They held small community meetings informing about the proposed programme and invited any families who met the inclusion criteria and keen to participate to give their names and details to a team member. Inclusion criteria were:

- at least one adolescent (aged 12 – 18 years) in the household
- permanent resident in the target community
- at least one parent/ carer and one adolescent available for weekly sessions.

A total of 239 of 245 families invited agreed to join the programme (97.6%). Based on team capacity there were 129 families recruited from Agra, 60 families from Robertsganj and 50 families from Khunti.

Data collection

In-depth interviews and focus group discussions (FGDs) were collected face-to-face in the community by KM, PS and PN in Hindi with 86 purposively sampled facilitators, parents and young people and continued until data saturation was reached. They lasted between 30 and 70 minutes and were recorded, translated and transcribed into English. Interview guides were developed by authors and piloted in the community. They asked about implementation processes, acceptability and feasibility as well as focus group discussions (FGD) with parents, adolescents and facilitators. Additional field notes and free text was recorded in coach and facilitator documents. Further data was collected during meetings with coaches which were conducted online, in the form of written notes taken during

For peer review only - http://bmjopen.bmj.com/site/about/guidelines.xhtml

meetings by a researcher not involved in meeting facilitation or participation (KM). Supplementary Table 2 provides an overview of the qualitative data collection.

Measures of fidelity

Coaches also filled a paper register at each site on the following measures:

- Facilitation and fidelity: 14 of 14 sessions self-reported by facilitators using a 5-point checklist where a score of 1 indicated Poor quality and a score of 5 indicated High quality. The following components of the intervention were assessed with this scale: physical exercise, emotional check in, main teaching topic, role play and completion of home activity.
- Facilitation and fidelity: 4 of 14 sessions for each Parwarish group were observed and measured by the coach, EHA project manager or a researcher who had all been trained to fill using the same criteria. Fidelity assessed the five aspects above and two additional aspects, namely: facilitator engagement, and completion of registers.
- Attendance of facilitators and coaches at trainings and coaching: attendance register.
- Attendance of parents and adolescents: attendance register
- Completion of assigned home activities: register.
- Home visits completed by facilitators: register.

Conceptual framework and analysis

This mixed methods study was analysed with a merging of two evaluation frameworks, namely process and feasibility evaluation, as we sought to understand how the intervention was delivered, and also whether it was feasible and acceptable. We followed the Medical Research Council framework for process evaluation of complex interventions examining how the intervention was delivered, and the emerging adaptations of the intervention related to context and actors (17). We also assessed feasibility which addresses facets of implementation that include practical, pragmatic and implementation processes used to deliver Parwarish programme to participants, adaptations to the Parwarish intervention required to increase engagement, understanding and relevance to diverse communities. Additionally, we examined acceptability and demand for Parwarish by asking participants whether they were comfortable with the group implementation processes and programme content (20).

Qualitative interviews and written documents were read and re-read and analysed with thematic analysis developed by Braun and Clarke (24). Four transcripts were inductively coded by PN and KM and these codes were reviewed to develop a coding framework, paying attention to implementation processes, feasibility and barriers and enablers of the intervention. Remaining transcripts were then coded with this framework by KM and PN,

For peer review only - http://bmjopen.bmj.com/site/about/guidelines.xhtml

who collaboratively developed categories and then themes, looking for patterns inherent in the data. Given the geographically dispersed field sites data was not member checked with parents and children in communities. Quantitative data was analysed descriptively and tabulated.

Patient and public involvement statement

Parents were involved in this study in three key ways. Firstly, parents were the impetus for this Parwarish intervention, having requested EHA team members in Dehradun, Agra and Khunti to support them to build their skills in raising young people. Secondly, parents were part of the process of adaptation of the intervention. A group of parents who resided in informal communities in Dehradun were consulted as “Experts by experience” in reviewing and suggesting contextually relevant adaptations of Parwarish. Thirdly, parents from intervention target communities were identified and trained as facilitators of this intervention and thus were also involved in recruiting participants, and collecting data linked to implementation required by facilitators such as the highlights and lowlights register. Parents in communities will also be involved in sharing findings at dissemination meetings planned after COVID-19 restrictions are lifted later in 2022 using community meetings and reviewing a plain-language Hindi summary of study findings.

Stance and ethics

This study was conducted by participant researchers who had all lived in India for at least 24 years and spoke fluent Hindi. KM, PS, PP (women) and PN (man) were employed by the implementers Emmanuel Hospital Association. KM (PhD) facilitated adaptation of PLH-Teens to Parwarish and led meetings with experts by experience. PS and PN (MPH and MSW) supported Parwarish implementation at a high level. PP (M.Med Sci) and IG (PhD) are women who work in India and Sweden respectively. Ethics approval was granted by the Institutional Ethics committee of EHA in Delhi for this study (Protocol 191) in February 2019.

Findings

Findings are presented under the broad headings of Context and Implementation proposed in the process evaluation of complex interventions guide of the MRC (17). We present broad features of the diverse contexts of each Parwarish implementation site. Evaluation of implementation presents quantitative and qualitative data summarising fidelity, dose and reach, followed by thematic analysis of the qualitative data presented under the domains of facilitation, community engagement and program infra-structure. Mechanisms of impact are described within the qualitative thematic analysis.

For peer review only - http://bmjopen.bmj.com/site/about/guidelines.xhtml

Context

Parwarish was implemented by teams from the Community development and Health programme (CHDP) of the Emmanuel Hospital Association in North India. The diverse study sites are summarised with a demographic profile of their three districts compared with national Indian data in Table 1. While all three sites report high proportions of young people and high rates of gender-based violence, the Jharkhand site is predominantly tribal and rural, while Agra is urban with a marked asymmetry in the sex ratio. Only half of women in the UP rural site of Sohbhadra are literate and the majority live rurally. Gender inequality is a key contextual factor that influenced implementation across all sites, and this is evidenced in Table 1. Asymmetries in the sex ratios of live births (fewer females) as well as the lower literacy rates of women underline the dominant patriarchy which is further discussed in qualitative findings of implementation.

Table 1. Socio-demographic profile of the three districts where Parwarish was implemented, with reference data for India nationally

Indicator	National - India	Agra City, Uttar Pradesh	Sonbhadra district, Uttar Pradesh	Khunti district, Jharkhand
Total population (million people)	1,200	4.41	1.86	0.53
% population rural	72.2	54.2	83.1	91.5
% population under 18 years (State)	34.9	33.7	33.7	33.5
% population Scheduled castes/Scheduled Tribes	25.2	22.6	14.9	77
Sex ratio (female to 1000 males)	940	868	918	997
Literacy (% literate female)	65.5	61.2	52.1	53.7
Literacy (% literate male)	82.1	80.6	74.9	74.1
Prevalence of gender-based violence (% at State level)	40	47	47	30 – 50

The context is further described by considering the socio-demographic profile of community participants profiled in Table 2. This demonstrates participants were highly disadvantaged and where over 80% of participants were from Scheduled Tribes or Scheduled castes, which are classifications assigned at birth and utilised by the Indian Government to indicate people groups who are structurally disadvantaged. Further indications of the social and economic disadvantage of participants are the fact that 55% of fathers and 75% of mothers hadn't completed primary schooling.

Table 2. Socio-demographic profile of Parwarish participants

For peer review only - http://bmjopen.bmj.com/site/about/guidelines.xhtml

(N=239 families)	Agra (%)	Robertsganj (%)	Khunti (%)	Parent	
	Adolescents/household			Mother	Father
**Female**	68 (52.7)	36 (60.0)	32 (64.0)	-	
**Age in years (Mean)**	13.3	13.3	14.0	36.8	40.2
**Caste (Indian census classification) :**					
Scheduled caste or tribes	110 (85.3)	51 (85)	50 (100)	199(83%)	
Other backward castes	19 (14.7)	9 (15)	-	37 (15%)	
General	-	-	-	3 (1.3%)	
**Religion:**					
Muslim	11 (8.5)	3 (5)	-	14 (6%)	
Hindu	117 (90.7)	57 (95)	3 (6)	167 (70%)	
Christian	-	-	21 (42)	29 (12%)	
Other religion	-	-	26 (52)	29 (12%)	
**Education:**					
Never been to school	31 (24.0)	-	-	135 (56%)	83 (35%)
Class 5 completed	46 (35.7)	32 (53.3)	23 (46)	48 (20%)	48 (20%)
Class 10 completed	44 (34.2)	48 (46.6)	20 (40)	43 (18%)	83 (35%)
Class 12 completed	8 (6.2)	-	3 (6)	2 (1%)	7 (3%)

Fidelity, dose and reach

Fidelity

Fidelity scores filled by facilitators and through each aspect of structured observation ranged from 70.0 – 82.0% (in a self-report five-item measure) and 75.7 – 78.6% (in a seven-item observed measure filled by coaches and researchers) suggesting fidelity to the intervention was moderate to good for all sites. Observers noted that facilitators initially omitted module components when pressed for time and read roleplays aloud rather than acting them out. Review of the value of role plays after Module 5 led to improved fidelity in delivery of this component of the intervention. The key omissions in fidelity to documentation was in failing to write detailed comments in registers to critically reflect on sessions (challenges, highlights) in the first 5-6 modules. After review and discussion with facilitators this improved. Session duration was a mean of 1.5 hours (Khunti), 2.2 hours (Agra) and 2.8 hours (Robertsganj) with sessions in Khunti reportedly shorter due to the pressure of agricultural work in the Jharkhand setting.

Dose and reach

There was 100% attendance in the Parwarish training by coaches and facilitators. Participant evaluations of Parwarish training were very positive, noting that there was opportunity to rehearse also put into practice components of the intervention. For example, training facilitators took turns leading the morning ‘emotional check-in’ and each also worked in pairs to develop their own household budget, a key output of later modules. Attendance of

For peer review only - http://bmjopen.bmj.com/site/about/guidelines.xhtml

facilitators and coaches at weekly coaching meetings was over 90%. Attendance and engagement of participants in Parwarish is summarised below in Table 3.

Participation was higher for women and adolescents than for men. Fathers explained their higher attendance in Agra due to encouragement by male facilitators so that just 13 of 129 fathers were classified as programme dropouts (missing four consecutive sessions) while few fathers attended in Agra and Khunti, reflecting dominant gender relations where men are expected to generate income.

For peer review only

For peer review only - http://bmjopen.bmj.com/site/about/guidelines.xhtml

Table 3. Summary of participation (attendance) in Parwarish

	Mean sessions attended (out of 14)	Mean number of home catch up sessions delivered (for those who missed a session) (out of 13)	Mean number of home activities completed
Mother	11.6	1.1	9.8
Father	5.8	3.3	4.8
Girls	11.2	1.0	10.5
Boys	11.1	1.1	10.2

Implementation

The seven themes that emerged are presented under the domains of relevance, acceptability and feasibility linked to implementation of the Parwarish intervention and are summarised in Table 4. A verbatim theme heading is provided to give greater texture to themes and is further illustrated with verbatim quotes taken from interviews and focus group discussions.

Table 4. Summary of key themes

Domain	Theme	Verbatim theme heading
Relevance	Facilitation by community members increased contextual validity of Parwarish	They can watch if our words ring true
	Implementation affected and was affected by gender relations	So, then we decided to take off our veils
Acceptability	Programme responsiveness to community needs increased participation	We talked about things I wanted to know
	Participation gathered momentum during implementation	I didn't plan to, but I ended up coming every week
Feasibility	Training promoted a strong core for implementation	We adjusted what we did depending on each group and each day
	Implementation was enhanced by technology	I liked seeing Whatsapp pictures to know what they were doing in Agra too
	Programme quality was strengthened by structured critical reflection	My husband and I sat and talked about what went well after facilitating each week

For peer review only - http://bmjopen.bmj.com/site/about/guidelines.xhtml

Relevance

"They can watch if our words ring true." – Facilitation by community members increased contextual validity of Parwarish Three coaches and 19 facilitators implemented Parwarish, and all (except one) were parents of an adolescent and lived in the target communities. Every facilitator reported that arguments with shouting or hitting occurred in their own household illustrating shared lived experience with participants. The 22 facilitators and coaches had a mean age of 39.9 years and 17 of 22 (77%) were from a Scheduled Caste or Tribe reflecting a similar demographic make-up to participants.

Parents and adolescents described a high level of trust with facilitators at two levels, firstly, through pre-existing relationships with the implementing teams; secondly, through facilitators who responded to their current needs and issues. One family described how the Parwarish facilitators supported them when their adolescent self-harmed and needed hospital care. A facilitator explains how their community base increased connection and trust below:

> *Because we live in this place anyone can see what work we do and how we do things all day long. If someone comes from outside, then people don't know how that person really lives but if we live in the same place, they can see if our words ring true in how we live. So, I think it is good to live in the same place as group members and also, I know the best way to explain things as I know about them and their lives.*

SSI, Facilitator, Agra

Programme facilitation by community members also presented challenges: community facilitators described feeling scrutinised by their neighbours in their own parenting and communication ('they watch to see if our words ring true') and some struggled to observe professional work ethics, E.g., to avoid gossip and breaches of confidentiality. Facilitators also described less engagement from groups most proximate to their own homes as described below:

> *"Being a facilitator in my own village and among people of my caste is more challenging ( ) as they don't take my words seriously and that reduces impact. But in other castes and communities people respond to my words positively."*

Facilitator, FGD, Robertsganj,

This mistrust was somewhat mitigated by the team taking pains to be punctual, reliable and prepared and increasing trust leading to increased participation as Parwarish progressed.

For peer review only - http://bmjopen.bmj.com/site/about/guidelines.xhtml

"So, then we decided to take off our veils" - Implementation affected and was affected by gender relations

The context of patriarchal gender relations which value men and disadvantage women impacted implementation in numerous ways. Implementers described reduced participation from men and boys who were less available due to income generation responsibilities. The lower rates of literacy for women also led to difficulties in identifying women as facilitators. Gender norms dictated women are commonly responsible for nurturing aspects of parenting meaning many households 'assigned' attendance of sessions to mothers.

A positive aspect of the gender norms which limited women from doing paid work in Agra was that mothers and daughters were available for Parwarish sessions during the day although in tribal Jharkhand both men and women work full time meaning reflecting tribal communities practice perhaps more equal gender relations than main-stream North India.

The sessions were facilitated in a way that sought to overcome typical age and gender related restrictions that might limit participation by young women, a key mechanism to support outcomes. Facilitators described how they sought participation from all with no 'right' or 'wrong' responses. A young woman described how this gave her courage to speak up:

> *Before this Parwarish course I would never give my opinion in front of adults I didn't know. But Anupriya (woman facilitator) would ask me to say what I thought and she said that there was no right or wrong answer so I could just give my opinion. Then I shared my opinion nearly every session and also I started to share my opinion at school.*

Young woman, Agra, FGD

Some resistance to traditional gender relations was evidenced through group participation which facilitators thought was triggered by both role modelling by facilitators as well as the process of equal valuing of participation by group members. For example, in Robertsganj and Agra initially there was widespread practice of *pardah* (a gendered practice prevalent through North India dictating men and women related to each other should not sit together and women should hide their faces from older male family members). After several sessions, facilitators and several women decided to challenge status quo gender relations by sitting beside males, and unveiling their faces as described:

> *"I decided it would be improper for men and women to sit together and see each other so I also wore pardah for first two sessions. But then I said I also couldn't understand what other women with faces covered were saying. So then we all decided to take our veils off and we were all showing our eyes.( )After two or three sessions in our facilitator group we also discussed the practice of purdah and use of*

For peer review only - http://bmjopen.bmj.com/site/about/guidelines.xhtml

face coverings and we decided we could make our own new rules for this group."

SSI, Female facilitator, Agra site

Acceptability

"We talked about things I wanted to know" - Programme responsiveness to community needs increased participation

The interactive format of Parwarish was identified as core to engagement, with sessions described as participatory and non-didactic. Facilitators described role-modelling the ideas as a key mechanism supporting outcomes, for example ensuring women and men participated in discussions while discussing the importance of family decision-making. Parents described sessions were responsive to their concerns as father describes below:

"I really liked coming to the group because we'd talk about things we wanted to know about and everyone gave me suggestions to solve all these problems my wife and I were having. Then after that we would also discuss about the parenting thing and that was interesting so then I decided to stay for all the sessions."

Father, FGD, Agra

Role plays also provided participants with 'positive' and 'negative' examples of how to engage in family negotiations and communications about relevant issues to this community, and were identified as a key mechanism to provoke thought and attitude change. A young women described how the role play approach helped her discuss her hopes for study with her parents:

I was really wanting to talk about how my parents had said I should stop attending school, but I didn't know how. Then in one of the role plays about Nagma she discusses with her mother what she really wants to do. I really loved that role play and then I could discuss with my mother about my hope that I want to do more school study

Teenage girl, FGD, Agra

However, facilitators also described challenges in using a non-didactic approach. They argued that eliciting responses made sessions lengthy and at times they then felt there was not time to complete roleplays. However, the home activities (such as praising children or developing a family budget) helped keep families engaged. One mechanism of learning was that young people described that they would revise and teach the subject matter to their siblings in their households. A mother also described how she would re-teach the weekly topic to remote family members by telephone as described below:

For peer review only - http://bmjopen.bmj.com/site/about/guidelines.xhtml

I really love the plays and stories in the Parwarish sessions and so once I brought along my sister Mira who was visiting from Delhi. Later Mira wanted to learn more so I would ring her after each session to tell her everything that happened. Then Mira's mother-in-law in Delhi had noticed Mira was being appreciative and praising her often which was a new thing, so her mother-in-law also wanted to learn about the session, so now Mira's whole household listens to the phone updates when I ring them each week.

Mother, FGD, Agra

'I didn't plan to but I ended up coming every week' - Participation gathered momentum during implementation Participants described multiple positive feedback loops that increased participation, engagement and ultimately outcomes as Parwarish progressed. Positive factors described included enjoyment of the group role plays and games, a sense of growing friendship with other participants, hearing others stories of similar struggles and appreciating new ideas in content of sessions. Parents described planning to attend just one session but then attending weekly as described below.

"I really liked the idea of learning more about how to parent my children but I felt like I didn't have time for this, so I thought I'll just go twice and stop. But after two sessions there were so many funny stories and examples from other parents having exactly the same problems as we have. So, then I decided that I would come every time.

Father, FGD, Agra

Later in Parwarish men described taking missing their daily wage work to participate and meeting at times of day they initially said were inconvenient. Momentum was also evident when adolescents, younger children and other ineligible community members requested if they could join the next programme.

Feasibility

Implementation of Parwarish in these low income settings was highly feasibility because the programme required minimal physical infrastructure: private homes and courtyards were used for group meetings although this led to challenges as homes were often too small for large groups impacting participation.

For peer review only - http://bmjopen.bmj.com/site/about/guidelines.xhtml

"We adjusted what we did depending on each group and each day" - Training promoted a strong core for implementation

Facilitators described that the in-depth training and coaching provided a clear core intervention on which they made pragmatic micro-adaptations as a more porous periphery to the intervention. Facilitators and coaches described applying Parwarish learnings to their own household leading to positive changes which increased their engagement and provided examples they used in group sessions. Micro-adaptations described by facilitators were in response to community requests or contextual and pragmatic such as changing the reducing group size from 15 to 10 families to fit with crowded urban settings or holding sessions in late evenings in villages where agricultural work dominated day-times.

"I liked looking at Whatsapp pictures to see what they were doing in Agra too" – Implementation was enhanced by technology

Coaches and facilitators identified social media platform Whatsapp as central for communications, coordination, supervision and feedback in real time. Photos and text were shared many times daily to coordinate and facilitate problem-solving between implementers. For example, photos of groups in cramped small rooms led to the solution proposed to meet in a more spacious outdoor courtyard. Coaches and coordinating team members described how Whatsapp messages provided encouragement and they identified a sense of competition and ability to showcase work as a mechanism that promoted them to work harder as facilitators. For example, the Agra team described how they felt support from others via Whatsapp when they had to run groups in a flooded courtyard so they persisted despite inconvenience. Messages also were used for coordination and data collection, such as to update baseline research forms filled, which motivated other sites, and increased compliance and participation for all teams.

For peer review only

For peer review only - http://bmjopen.bmj.com/site/about/guidelines.xhtml

“My husband and I sat and talked about what went well after leading each week” - Programme quality was strengthened by structured critical reflection Parwarish implementation used formats and structures developed in PLH-Teen in South Africa to trigger critical reflection and improve intervention quality. For example, each facilitator filled a format after sessions describing highlights (what had gone well) as well as challenges (what didn’t go well), and their responses to observations and these were discussed with their coach. A fortnightly meeting between coaches in the three sites, the coordination team and the PLH-Teens support team used Skype group conversations with a translator increased the quality of coordination, reporting and real-time responsiveness and reduced travel.

Facilitators described how the formational Parwarish training changed their own attitudes to gender equality in marriage relations and adult-child (age) hierarchies. Coaches also described the fortnightly reports as improving their own group facilitation prompted them to help facilitators solve their own problems. An example of attitude change leading to behaviour change is described below:

> *“(In our weekly facilitator meeting) Sushila described how she had never asked her three sons to help in the kitchen, but the other facilitators described how their sons would make chapatis and all. ( ) So then Sushila told us how once getting late and so she had rung her son to ask him to wash dishes and he did that. She said it was a very positive experience.”*

Discussion

This process evaluation shows good acceptability and relevance for the Parwarish intervention evidenced by high levels of attendance, participation and engagement by parents and adolescents in three deprived socio-economic settings in India, and implementation that was feasible in disadvantaged urban and rural settings in central and North India.

Enrolment and attendance rates for fathers and mothers in Parwarish were significantly higher than in PLH-Teens implementations in South Africa, Philippines and Sudan (12, 25, 26). Factors likely to have increased attendance include context-relevant adaptations by the community-based facilitators and the pre-existing strong relationships and community development work of implementing teams. The novelty of a first-ever research project with daily Whatsapp group reporting across sites perhaps added a competitive component that promoted greater activity among facilitators (27).

For peer review only - http://bmjopen.bmj.com/site/about/guidelines.xhtml

The results demonstrate Parwarish was feasibly implemented by lay community facilitators and coaches. This has positive implications for scaling across South Asia and beyond: implementation by local people receiving modest remuneration in meeting spaces provided by the community makes this a relatively low-cost intervention (25). However, while the program is likely to be feasible and acceptable in other disadvantaged settings in South Asia, the high level of commitment by facilitators (for example, being willing to conduct meetings at night in Jharkhand communities) may be harder to replicate. Implementers also underlined the value of structured, high-intensity program buttressing to increase fidelity and quality of Parwarish, which was also identified as central in PLH-Teens implementation (14, 22, 23) and other community-based group interventions in South Asia (28-30).

Implementers and facilitators described the India-specific adaptation of PLH-Teens as relevant and acceptable in two key ways: firstly, the curricular adaptations that were superficial (e.g. names of characters in role plays) were appropriate and felt recognisable, while deeper changes, such as implementation by married couples and holding group meetings in the private homes / courtyards in Uttar Pradesh sites made Parwarish feasible, culturally appropriate and easy-to-engage-with in these settings(25, 26).

Secondly, relevance and acceptability were increased by deployment of trained community members facilitating groups, using their in-depth knowledge of the daily lives, culture and language (ie. context) of participants. Parwarish facilitators used local dialects in facilitation and gave examples familiar to participants from shared common knowledge, adjusting logistics and roleplays in each setting to maximise participation. Implementers described the importance of trusting the innate contextual knowledge of facilitators (31) and the value of facilitators discussing their proposed adaptations to the programme with PLH-Teens coaches to ensure the core was followed but in locally acceptable ways. Facilitators described a range of continuous micro-adaptations, a feature key to successful interventions in other low- and middle-income settings (28, 32, 33). At the same time, some of the changes such as meeting at night perhaps reduced visual learning opportunities (for example from role plays) as there was no electricity at night.

The effectiveness of Parwarish was enhanced through the program supports of coaching to increase facilitator competency, also a key theme in other PLH programmes (23, 25, 26). Strategies increasing intervention fidelity and quality included observation and feedback by coaches, weekly meetings, checklists and fortnightly ‘coach the coaches’ sessions. The fidelity to the programme was acceptable but required structured feedback to solved problems such as meeting timing and needed attentive discussion to address more challenging aspects like acting out role plays which were omitted due to discomfort in initial modules. The need for a balance between fidelity and ‘fit’ with the local context is widely described(23, 34, 35) and in these findings there were some compromises in fidelity to ‘fit’ the community needs.

For peer review only - http://bmjopen.bmj.com/site/about/guidelines.xhtml

While participation of fathers in Parwarish was greater than has occurred in other PLH-Teens settings, men's attendance was still significantly less than women's. Men's participation in any family linked intervention is critical as they typically control financial decisions and dominate family decision-making in Indian households(36, 37). Strategies to increase participation of men include including men as facilitators, ensuring meetings are held at times when men can attend, and developing momentum for men's participation by engaging with men who are 'positive deviants'(36). India is among the world's bottom five countries in the health and survival category of the Global gender gap index meaning there are large gender disparities that disadvantage women (6)**.** Parwarish held transforming gender relations as core to process, content and implementation with men and women seated together in a group which already confronted the predominant uni-sex socailising and gender relations, an approach endorsed elsewhere in North India (36).

Gender relations influenced participation, for example decreasing attendance of fathers and sons, because of gender role expectations to generate income (12, 25, 26). There were also proportionately more girls among participating adolescents, perhaps reflecting their lower priority for school attendance and employment (6). Parwarish enabled new attitudes and transformations to gender relations. For example, the Agra and Robertsganj groups' decision to not observe purdah when in group meetings was key to the quality and quantity of dialogue in the group meetings and went on to lead to other outcomes where women described increased autonomy in decision-making and increased participation in family financial decisions. This early outcome generated a sense of entrenched behaviours linked to harsh parenting also being open to negotiation and change, and encouraged facilitators, mothers and young women, which then increased their engagement and participation; this positive feedback loop has been identified as important in other gender transformative interventions (36). Addressing deeply embedded gender relations of patriarchy prevalent across South Asia has no quick fix solution, and further work on gender transformative interventions is urgently needed in communities, schools, and national policies across India to bring greater gender equality which can benefit both women and men(36).

Parwarish groups used face-to-face trainings and meetings, and both facilitator manuals and monitoring formats were paper-based, yet implementation was strengthened through technological applications and mobile phones. In India there are over 1 billion users of mobile phones (38) and 400 million people using the application Whatsapp(39). The benefits of Whatsapp which is familiar (widely used by peers), free and synchronous and easy to use to support problem solving and quality of community health programme implementation in low resourced settings has been described by others (27, 40) and further realised in the COVID-19 era (41, 42).Facilitators and coaches described three new ways technology enhanced their work: firstly in Whatsapp group communications between coaches and facilitators allowing sharing of pictures and video calls which strengthened monitoring and

For peer review only - http://bmjopen.bmj.com/site/about/guidelines.xhtml

program quality. Secondly for coordination with implementers in New Delhi, with audio and visual facets increasing the ability to communicate the challenges and opportunities in field settings;and thirdly, in the use of Skype conversations for ‘coach the coaches’ sessions between field sites, New Delhi and South Africa. .

Methodological considerations

There are some important limitations to this study. First, researchers were involved in higher level implementation of this project (PS and PN) which could have led to social desirability bias in responses from both facilitators and Parwarish participants. However a benefit of including implementers as researchers was that PS, KM and PN had an in-depth understanding of factors influencing implementation as well as contextual challenges. Second, the evaluation did not include a control group and was evaluated immediately after implementation limiting knowledge about sustainability of changes. Third, while researchers visited implementation sites several times, no researchers resided in intervention communities which reduced their contextual understanding of the relevance and acceptability of the implementation process. There were important strengths in the study design: First, it reflected the ‘real-world’ allowing iterative micro-adaptations of the intervention to make it optimally relevant and acceptable in the community. Secondly, implementation by community members as facilitators, with groups housed in communities using local homes to host groups and a modest budget in three diverse sites provides a model for implementation that can be generalised for implementation and scaling by others working among people from low-income communities across South Asia.

Conclusions

This evaluation demonstrates the Parwarish intervention as acceptable, relevant and feasible in an urban slum and a rural agricultural town in North India and a remote tribal region of Central India. In a vast country which has seen almost no examples of programmes seeking to increase positive parenting and reduce family violence, this pilot study shows the implementation process was engaging, feasible and acceptable in communities, which paves the way for larger-scale implementation in other settings in India and South Asia.

Ethics statement

The Emmanuel Hospital Association Institutional Ethics committee, New Delhi approved this study (Protocol 191) in March 2019.

For peer review only - http://bmjopen.bmj.com/site/about/guidelines.xhtml

Contributor ship statement

KM conceived of the study, designed it with PS and IG and wrote the first draft, PN collected data with PS, and completed analysis with KM, PP and IG, and all authors contributed to multiple drafts of the paper.

Funding

This work was supported by the Swedish Research Council: 2017-05421.

Competing interests

All authors declare they had no competing interests in the implementation and evaluation of this intervention and study.

Data sharing

No additional data available

References

1. Patel R, Gupte SS, Srivastava S, Kumar P, Chauhan S, Govindu MD, et al. Experience of gender-based violence and its effect on depressive symptoms among Indian adolescent girls: Evidence from UDAYA survey. PLoS One. 2021;16(3):e0248396.
2. Deb S, Modak S. Prevalence of violence against children in families in Tripura and its relationship with socio-economic factors. J Inj Violence Res. 2010;2(1):5-18.
3. Daral S, Khokhar A, Pradhan S. Prevalence and Determinants of Child Maltreatment among School-Going Adolescent Girls in a Semi-Urban Area of Delhi, India. J Trop Pediatr. 2016;62(3):227-40.
4. Ellsberg M, Arango DJ, Morton M, Gennari F, Kiplesund S, Contreras M, et al. Prevention of violence against women and girls: what does the evidence say? The Lancet. 2015;385(9977):1555-66.
5. Norman RE, Byambaa M, De R, Butchart A, Scott J, Vos T. The long-term health consequences of child physical abuse, emotional abuse, and neglect: a systematic review and meta-analysis. PLoS Med. 2012;9(11):e1001349.
6. World Economic Forum. Global Gender Gap report 2021. Geneva: World Economic Forum; 2021.
7. Knerr W, Gardner F, Cluver L. Improving positive parenting skills and reducing harsh and abusive parenting in low-and middle-income countries: a systematic review. Prevention science. 2013;14(4):352-63.
8. de Wit EE, Chakranarayan C, Bunders-Aelen JF, Regeer BJ. Learning about parenting together: a programme to support parents with inter-generational concerns in Pune, India. Contemp Fam Ther. 2018;40(1):68-83.
9. Isaac R, Annie I, Prashanth H. Parenting in India. Parenting across cultures: Springer; 2014. p. 39-45.
10. Brar P, Dworkin J, Jang J. Association of parenting with sexual attitudes and behaviors of college students in India. Sexuality & Culture. 2018;22(4):1037-53.

For peer review only - http://bmjopen.bmj.com/site/about/guidelines.xhtml

For peer review only

11. Pedersen GA, Smallegange E, Coetzee A, Hartog K, Turner J, Jordans MJ, et al. A systematic review of the evidence for family and parenting interventions in low-and middle-income countries: child and youth mental health outcomes. Journal of Child and Family Studies. 2019:1-20.
12. Cluver L, Meinck F, Yakubovich A, Doubt J, Redfern A, Ward C, et al. Reducing child abuse amongst adolescents in low- and middle-income countries: A pre-post trial in South Africa. BMC Public Health. 2016;16(1):567.
13. Cluver LD, Meinck F, Steinert JI, Shenderovich Y, Doubt J, Herrero Romero R, et al. Parenting for Lifelong Health: a pragmatic cluster randomised controlled trial of a non-commercialised parenting programme for adolescents and their families in South Africa. BMJ Glob Health. 2018;3(1):e000539.
14. Cluver L, Meinck F, Shenderovich Y, Ward CL, Romero RH, Redfern A, et al. A parenting programme to prevent abuse of adolescents in South Africa: study protocol for a randomised controlled trial. Trials. 2016;17(1):328.
15. Cluver L, Lachman J, Ward C, Gardner F, Petersen T, Meinck F, et al. Development of a parenting programme to prevent abuse of adolescents in South Africa: Findings from a pre-post pilot study. Research on Social Work Practice. 2016.
16. Lachman JM, Cluver LD, Boyes ME, Kuo C, Casale M. Positive parenting for positive parents: HIV/AIDS, poverty, caregiver depression, child behavior, and parenting in South Africa. AIDS Care. 2014;26(3):304-13.
17. Moore GF, Audrey S, Barker M, Bond L, Bonell C, Hardeman W, et al. Process evaluation of complex interventions: Medical Research Council guidance. BMJ : British Medical Journal. 2015;350:h1258.
18. Oakley A, Strange V, Stephenson J, Forrest S, Monteiro H. Evaluating processes: a case study of a randomized controlled trial of sex education. Evaluation. 2004;10(4):440-62.
19. Brand SL, Quinn C, Pearson M, Lennox C, Owens C, Kirkpatrick T, et al. Building programme theory to develop more adaptable and scalable complex interventions: Realist formative process evaluation prior to full trial. Evaluation. 2019;25(2):149-70.
20. Bowen DJ, Kreuter M, Spring B, Cofta-Woerpel L, Linnan L, Weiner D, et al. How we design feasibility studies. Am J Prev Med. 2009;36(5):452-7.
21. Colucci E, Hassan G. Prevention of domestic violence against women and children in low-income and middle-income countries. Current Opinion in Psychiatry. 2014;27(5):350-7.
22. Lachman JM, Cluver L, Ward CL, Hutchings J, Mlotshwa S, Wessels I, et al. Randomized controlled trial of a parenting program to reduce the risk of child maltreatment in South Africa. Child Abuse Negl. 2017;72:338-51.
23. Lachman JM, Kelly J, Cluver L, Ward CL, Hutchings J, Gardner F. Process evaluation of a parenting program for low-income families in South Africa. Research on Social Work Practice. 2018;28(2):188-202.
24. Braun V, Clarke V. Using thematic analysis in psychology. Qualitative research in psychology. 2006;3(2):77-101.
25. Janowski RK, Wessels I, Bojo S, Monday F, Maloney K, Achut V, et al. Transferability of evidence-based parenting programs to routine implementation in postconflict South Sudan. Research on Social Work Practice. 2020;30(8):858-69.
26. Alampay LP, Lachman JM, Landoy BV, Madrid BJ, Ward CL, Hutchings J, et al. Preventing Child Maltreatment in Low‐ and Middle-Income Countries: Parenting for Lifelong Health in the Philippines. Developmental science and sustainable development goals for children and youth: Springer; 2018. p. 277-93.
27. Henry JV, Winters N, Lakati A, Oliver M, Geniets A, Mbae SM, et al. Enhancing the Supervision of Community Health Workers With WhatsApp Mobile Messaging: Qualitative Findings From 2 Low-Resource Settings in Kenya. Global Health: Science and Practice. 2016;4(2):311-25.
28. Khan MN, Hamdani SU, Chiumento A, Dawson K, Bryant RA, Sijbrandij M, et al. Evaluating feasibility and acceptability of a group WHO trans-diagnostic intervention for women with common

For peer review only - http://bmjopen.bmj.com/site/about/guidelines.xhtml

mental disorders in rural Pakistan: a cluster randomised controlled feasibility trial. Epidemiology and psychiatric sciences. 2017:1-11.
29. Leventhal KS, DeMaria LM, Gillham JE, Andrew G, Peabody J, Leventhal SM. A psychosocial resilience curriculum provides the “missing piece” to boost adolescent physical health: A randomized controlled trial of Girls First in India. Soc Sci Med. 2016;161:37-46.
30. Leventhal KS, Gillham J, DeMaria L, Andrew G, Peabody J, Leventhal S. Building psychosocial assets and wellbeing among adolescent girls: A randomized controlled trial. J Adolesc. 2015;45:284-95.
31. Shen J, Yang H, Cao H, Warfield C. The fidelity—Adaptation relationship in non-evidence-based programs and its implication for program evaluation. Evaluation. 2008;14(4):467-81.
32. Nakkash RT, Alaouie H, Haddad P, El Hajj T, Salem H, Mahfoud Z, et al. Process evaluation of a community-based mental health promotion intervention for refugee children. Health Educ Res. 2012;27(4):595-607.
33. Sharma S, Adetoro OO, Vidler M, Drebit S, Payne BA, Akeju DO, et al. A process evaluation plan for assessing a complex community-based maternal health intervention in Ogun State, Nigeria. BMC Health Serv Res. 2017;17(1):238.
34. Castro FG, Barrera M, Martinez CR. The cultural adaptation of prevention interventions: resolving tensions between fidelity and fit. Prevention Science. 2004;5(1):41-5.
35. Limbani F, Goudge J, Joshi R, Maar MA, Miranda JJ, Oldenburg B, et al. Process evaluation in the field: global learnings from seven implementation research hypertension projects in low-and middle-income countries. BMC Public Health. 2019;19(1):953.
36. Freudberg H, Contractor S, Das A, Kemp CG, Nevin PE, Phadiyal A, et al. Process and impact evaluation of a community gender equality intervention with young men in Rajasthan, India. Culture, health & sexuality. 2018:1-16.
37. Theobald S, Morgan R, Hawkins K, Ssali S, George A, Molyneux S. The importance of gender analysis in research for health systems strengthening. Health Policy Plan. 2017;32(suppl_5):v1-v3.
38. Telecom regulatory authority of India. India’s active mobile users reached 1,026.37 million. New Delhi: Telecom regulatory authority of India; 2019.
39. WhatsApp may soon touch 500 million users in India despite new privacy policy, claims report [press release]. New Delhi: India Today2021.
40. Perry H, Crigler L, Gergen J. Supervision of community health workers. In: Perry H, Crigler L, editors. Developing and strengthening community health worker programs at scale - aA reference guide and case studies for program managers and policymakers Baltimore, MD: MCHIP, USAID. Jhpiego Corporation; 2013.
41. Ajisegeri W, Odusanya O, Joshi R. Covid-19 outbreak situation in Nigeria and the need for effective engagement of community health workers for epidemic response. Global Biosecurity. 2020;1(4).
42. How WhatsApp became the tool of choice for WHO's COVID-19 messaging [press release]. 2020.

For peer review only

For peer review only - http://bmjopen.bmj.com/site/about/guidelines.xhtml

Supplementary files

Supplementary file 1: Overview of Parwarish curriculum content

Session	Key content
1	Introducing the programme and setting participant goals
2	The value of spending time together to build a positive parent – child relationship
3	Why praise and appreciation build connection – and practicing praise
4	Identifying and talking about emotions in the family
5	Managing anger without hurting others
6	Identifying problems and following steps for problem solving
7/8	The value of saving money and how to make a budget
9	Addressing problems without conflict (2 sessions)
10	Developing family rules and routines to reduce conflict and have fun
11	Ways to save money and make a family savings plan
12	Identifying risks and ways to keep safe in the community
13	How to manage in crisis – safeguards and strategies
14	Getting help and support beyond your household: widening the circle

Supplementary file 2: Qualitative data collection overview

**Participants**	**Data collection**
Facilitators at each site	3 FGD with facilitators (n= 22 people)
Parents and adolescent participants in Parwarish	4 FGD with parents (n = 29) and 4 FGD with adolescents (n= 23)
Online coach session with 3 site coaches and implementation coordinator and researcher	Detailed notes of online session (total 11 pages of typed text)
Facilitators (community-based pairs)	6 individual interviews
Program implementers (Delhi and Agra based)	3 individual interviews
Coaches (located at each of the three field sites)	3 individual interviews
Observations of training, group facilitation in field and coaching sessions	Field notes (total 14 pages of typed text)
Fortnightly logbook documenting highlights and challenges on sessions filled by coaches and shared fortnightly	Fortnightly logbook shared as an email with PN and KM and South African CWB coach
Total number of people contributing to qualitative data	N=86

For peer review only

COREQ (COnsolidated criteria for REporting Qualitative research) Checklist

A checklist of items that should be included in reports of qualitative research. You must report the page number in your manuscript where you consider each of the items listed in this checklist. If you have not included this information, either revise your manuscript accordingly before submitting or note N/A.

Topic	Item No.	Guide Questions/Description	Reported on Page No.
**Domain 1: Research team and reflexivity**			
Personal characteristics			
Interviewer/facilitator	1	Which author/s conducted the interview or focus group?	
Credentials	2	What were the researcher's credentials? E.g. PhD, MD	
Occupation	3	What was their occupation at the time of the study?	
Gender	4	Was the researcher male or female?	
Experience and training	5	What experience or training did the researcher have?	
Relationship with participants			
Relationship established	6	Was a relationship established prior to study commencement?	
Participant knowledge of the interviewer	7	What did the participants know about the researcher? e.g. personal goals, reasons for doing the research	
Interviewer characteristics	8	What characteristics were reported about the inter viewer/facilitator? e.g. Bias, assumptions, reasons and interests in the research topic	
**Domain 2: Study design**			
Theoretical framework			
Methodological orientation and Theory	9	What methodological orientation was stated to underpin the study? e.g. grounded theory, discourse analysis, ethnography, phenomenology, content analysis	
Participant selection			
Sampling	10	How were participants selected? e.g. purposive, convenience, consecutive, snowball	
Method of approach	11	How were participants approached? e.g. face-to-face, telephone, mail, email	
Sample size	12	How many participants were in the study?	
Non-participation	13	How many people refused to participate or dropped out? Reasons?	
Setting			
Setting of data collection	14	Where was the data collected? e.g. home, clinic, workplace	
Presence of non-participants	15	Was anyone else present besides the participants and researchers?	
Description of sample	16	What are the important characteristics of the sample? e.g. demographic data, date	
Data collection			
Interview guide	17	Were questions, prompts, guides provided by the authors? Was it pilot tested?	
Repeat interviews	18	Were repeat inter views carried out? If yes, how many?	
Audio/visual recording	19	Did the research use audio or visual recording to collect the data?	
Field notes	20	Were field notes made during and/or after the inter view or focus group?	
Duration	21	What was the duration of the inter views or focus group?	
Data saturation	22	Was data saturation discussed?	
Transcripts returned	23	Were transcripts returned to participants for comment and/or	

For peer review only

Topic	Item No.	Guide Questions/Description	Reported on Page No.
		correction?	
**Domain 3: analysis and findings**			
Data analysis			
Number of data coders	24	How many data coders coded the data?	
Description of the coding tree	25	Did authors provide a description of the coding tree?	
Derivation of themes	26	Were themes identified in advance or derived from the data?	
Software	27	What software, if applicable, was used to manage the data?	
Participant checking	28	Did participants provide feedback on the findings?	
Reporting			
Quotations presented	29	Were participant quotations presented to illustrate the themes/findings? Was each quotation identified? e.g. participant number	
Data and findings consistent	30	Was there consistency between the data presented and the findings?	
Clarity of major themes	31	Were major themes clearly presented in the findings?	
Clarity of minor themes	32	Is there a description of diverse cases or discussion of minor themes?	

Developed from: Tong A, Sainsbury P, Craig J. Consolidated criteria for reporting qualitative research (COREQ): a 32-item checklist for interviews and focus groups. *International Journal for Quality in Health Care*. 2007. Volume 19, Number 6: pp. 349 – 357

Once you have completed this checklist, please save a copy and upload it as part of your submission. DO NOT include this checklist as part of the main manuscript document. It must be uploaded as a separate file.

For peer review only
